# Genome-Wide Identification, Characterisation and Phylogenetic Analysis of 52 Striped Catfish (*Pangasianodon hypophthalmus*) ATP-Binding Cassette (ABC) Transporter Genes

**DOI:** 10.21315/tlsr2022.33.2.12

**Published:** 2022-07-15

**Authors:** Leonard Whye Kit Lim, Hung Hui Chung, Han Ming Gan

**Affiliations:** 1 Faculty of Resource Science and Technology, Universiti Malaysia Sarawak, 94300 Kota Samarahan, Sarawak, Malaysia; 2 GeneSEQ Sdn Bhd, Bukit Beruntung, 48300 Rawang, Selangor, Malaysia; 3 Centre for Integrative Ecology, School of Life and Environmental Sciences, Deakin University, Geelong, Victoria, Australia

**Keywords:** Striped Catfish, Genome-Wide, ABC Transporters, Motif Analysis, Phylogeny, Ikan Keli Berjalur, Seluruh Genom, Pengangkut ABC, Analisis Motif, Filogeni

## Abstract

The *Pangasianodon hypophthalmus* (striped or tra catfish) is a Pangasiidae family member famous for its high unsaturated fatty acid content flesh. This riverine catfish can breathe in the air unlike the channel catfish. One of the most well-known ecotoxicological protein superfamily, the ATP-binding cassette (ABC) transporters, has been characterised in channel catfish through a genome-wide approach. Therefore, it is interesting to unearth these proteins within the striped catfish genome for a comprehensive comparison across all catfishes available. A total of 52 ABC transporters were discovered from the striped catfish genome. The motif analysis has unconcealed various unshared characteristics of some catfishes. The phylogenetic analysis has evidenced its effectiveness in the successful annotations of these transporter proteins. Duplicated genes such as ABCA1, ABCB3, ABCB6, ABCC5, ABCD3, ABCE1, ABCF2 as well as ABCG2 were uncovered within the striped and channel catfish genomes. This entire set of ABC transporters yields precious genomic data for future ecotoxicological, biochemical and physiological research in striped catfish.

## Abstract (ms)

*Pangasianodon hypophthalmus* (ikan keli bergaris atau tra) ialah ahli keluarga Pangasiidae yang terkenal dengan kandungan asid lemak tak tepu yang tinggi. Ikan keli sungai ini dapat bernafas di udara tidak seperti ikan keli terusan. Salah satu keluarga besar protein ekotoksikologi yang paling terkenal, pengangkut kaset ATP (ABC), telah dicirikan dalam ikan keli saluran melalui pendekatan seluas genom. Oleh itu, adalah menarik untuk menggali protein ini di dalam genom ikan keli bergaris untuk perbandingan menyeluruh antara semua ikan keli yang ada. Sebanyak 52 pengangkut ABC ditemui dari genom ikan keli berjalur. Analisis motif telah membongkar pelbagai ciri yang tidak dikongsi antara beberapa ikan keli. Analisis filogenetik telah membuktikan keberkesanannya dalam anotasi protein transporter yang berjaya. Gen pendua seperti ABCA1, ABCB3, ABCB6, ABCC5, ABCD3, ABCE1, ABCF2 dan juga ABCG2 ditemui dalam genom ikan keli berjalur dan saluran. Keseluruhan rangkaian pengangkut ABC ini menghasilkan data genomik yang berharga untuk penyelidikan ekotoksikologi, biokimia dan fisiologi masa depan pada ikan keli bergaris.

**Highlights**

- A total of 52 ABC transporters were discovered from the striped catfish genome.
- Duplicated genes such as ABCA1, ABCB3, ABCB6, ABCC5, ABCD3, ABCE1, ABCF2 as well as ABCG2 were uncovered within the striped and channel catfish genomes.
- The motif analysis has revealed several exclusive characteristics of some catfishes which provides unique genomic landscape for future ecotoxicological, biochemical and physiological researches.

## INTRODUCTION

The striped catfish (*Pangasianodon hypophthalmus*), also known as the tra catfish, is a freshwater riverine belonging to the Pangasiidae family. This catfish is a facultative air-breather whereby it utilises its swim bladder as an air-breathing organ, unlike the channel catfish (*Ictalurus punctatus*) that is unable to breathe in the air (Ma *et al*. 2021). This fish was first cultured widely in Vietnam and was later introduced to countries like Malaysia, China, Philippines, Singapore and India (Gao *et al*. 2021). The tra catfish is highly appreciated for its unsaturated fatty acids content (Kim *et al*. 2018). Around 59% of the striped catfish weight is contributed by the crude fat found within the head and flab parts. Surprisingly, the unsaturated fatty acids in the crude fat encompasses a sum of 60% (Gao *et al*. 2021). This economically important fish should be researched on immediately, especially on its detoxification aspect (Nguyen *et al*. 2016), apart from its growth and nutrient improvement aspects. Hence, one of the most renowned detoxification efflux pump orchestrators, namely the ATP-binding cassette (ABC) transporters, was selected as the main focus of this work.

The ABC transporters are present in all organisms including human and microorganisms, making up one of the largest protein families known to date (Liu *et al*. 2013). These transporters translocate substrates such as toxins, xenobiotics, ions and metabollites across membranes via their conserved nucleotide-binding domains (NBDs) and transmembrane domains (TMDs). There are six major subfamilies encompassing protein members from ABCA to ABCH, each of them is associated with various essential biological functions (Andersen *et al*. 2015; Guo *et al*. (2015a); Park *et al*. 2016). The *ABCH1* gene is exclusively absent in mammals, but present in insects, mold, fish and other vertebrates (Popovic *et al*. 2010). The subfamily members of ABCA, ABCB and ABCC are full transporters whereas the subfamily members of ABCD, ABCE/ABCF and ABCG/ABCH contain only half transporters. These proteins have been proven as pivotal agrochemical and ecotoxicological markers in fishes closely related to striped catfish, namely zebrafish, Sarawak rasbora, common carp and channel catfish, in assessing the water quality of a natural habitat (Popovic *et al*. 2010; Liu *et al*. 2013; 2016; Lim *et al*. 2018; Yeaw *et al*. 2020; Md Yusni *et al*. 2020; Lai *et al*. 2021; Lim *et al*. 2021a). By elucidating the functional roles of these proteins in indicator fish species like the tra catfish, we can finally decipher the underlying detoxification mechanism that is extremely useful in aiding us to further enhance and strengthen the immunity system of this valuable aquaculture fish in order to minimise economical loss due to infections in the fish farming industry (Chung *et al*. 2020; Lau *et al*. 2021a; 2021b; Lim *et al*. 2021b). Therefore, in this study, we aimed to unearth and characterise the 52 genome-wide ABC proteins of striped catfish in hope to further enrich the detoxification database of this catfish to support future metabolism studies and conservation endeavors.

## METHODOLOGY

### Transcriptome Sequencing and Genome-wide Identification of ABC Proteins

The juvenile striped catfish (body length: 5 cm) was sampled from an aquaculture centre in Kuching, Sarawak, Malaysia (GPS coordinates: 1.4700676917664783, 110.33115299732843). The fish was deposited as voucher specimen (voucher ID: ASD03019) in the fish museum situated inside the Faculty of Resource Science and Technology, Universiti Malaysia Sarawak. The euthanisation of the fish was conducted in compliance to the guidelines and permissions granted by the Animal Ethics Committee of Universiti Malaysia Sarawak with permit number UNIMAS/TNC(PI)-04.01/06-09(17). The total RNA of striped catfish was extracted from the muscle tissues using Wizol TriZol-like reagent (WizBio, Republic of Korea) according to the manufacturer’s protocol. The purified total RNA was then subjected to mRNA enrichment process employing poly-T magnetic bead (NEB, England). Next, the library was prepared from the enriched mRNAs using NEB Ultra II RNA library (NEB, England). Transcriptome sequencing was then performed on an Illumina NovaSeq6000 (2 x 150 bp). Transcriptome assembly statistics were generated using QUAST (Gurevich *et al*. 2013). The genome-wide ABC protein sequences of channel catfish (Liu *et al*. 2013) were retrieved from the public GenBank database, to be used as a reference. The genome scaffolds of striped catfish were blasted against the reference ABC protein sequences of channel catfish via DIAMOND (Buchfink *et al*. 2015) with the cutoff value set at above 70% similarity. Additional protein BLAST searches (>70% identity cutoff value) were also conducted across ABC sequences of other catfishes (Asian redtail catfish, yellowhead catfish, black bullhead catfish, channel catfish, walking catfish, Chinese large-mouth catfish and giant devil catfish) from the GenBank database to look for ABC proteins not present in channel catfish.

### ABC Protein Characterisation and Phylogenetic Analysis

The ABC proteins of striped catfish were first subjected to amino acid length, molecular weight and theoretical isoelectric point (pI) predictions via Sequence Manipulation Suite (Stothard 2000). Next, the subcellular localisation was predicted employing CELLO server version 2.5 (Yu *et al*. 2006). The domain structure prediction was conducted utilising SMART tool (Letunic *et al*. 2021). A motif analysis was performed for each ABC subfamily using MEME suite tool (Bailey *et al*. 2015). Motif identity was determined through NCBI conserved domain search (Yang *et al*. 2020) and ScanProsite (De Castro *et al*. 2006). The ABC proteins were aligned before undergoing a model test using MEGA X (Kumar *et al*. 2018). The selected model is the JTT+I+G (Jones-Taylor-Thornton matrix with consideration of invariant sites [+I] and gamma distribution for modeling rate heterogeneity [+G]) model. Phylogenetic tree was constructed using MEGA X (Kumar *et al*. 2018) based on all ABC proteins across different catfishes besides the individual trees plotted representing each ABC subfamily, with 1000 bootstrap replications and maximum likelihood criterion.

## RESULTS AND DISCUSSION

### Transcriptome Sequencing, Motif and Phylogenetic Analysis of ABC Transporters in Striped Catfish

The transcriptome of the striped catfish was sequenced (GenBank SRA accession number: SRR17621361) and the assembly statistics were enumerated using QUAST (Gurevich *et al*. 2013). A total of 154,159 contigs were unearthed from the striped catfish genome (Table 1). About 31.17% of them have length of at least 1000 bp. Approximately 2.36% and 0.08% of them have at least 5000 bp and 10,000 bp, respectively. No contigs were found to have length larger than 25,000 bp and 50,000 bp as the largest length was 17,647 bp. The total length of contigs is 143,584,872 bp with 47.92% of them having more than 500 bp in length. The total GC content recorded was 44.52%. The N50 and N75 of the tra catfish transciptome assembly are 2,729 bp and 1,594 bp, respectively. This indicates that the median value of contig length is around 2,729 bp, a skew towards shorter contigs was observed for this assembly. This value is much lower to that determined by Kim *et al*. (2018) where the N50 calculated was 14.29 Mbp. On the other hand, the L50 and L75 were determined at 17,010 and 34,065, respectively. This means that almost 11% of total contigs (17,010 contigs) encompass half of the total base content of the tra catfish transcriptome assembly. No N nucleotide was detected per 100 kbp.

**Figure 1:**
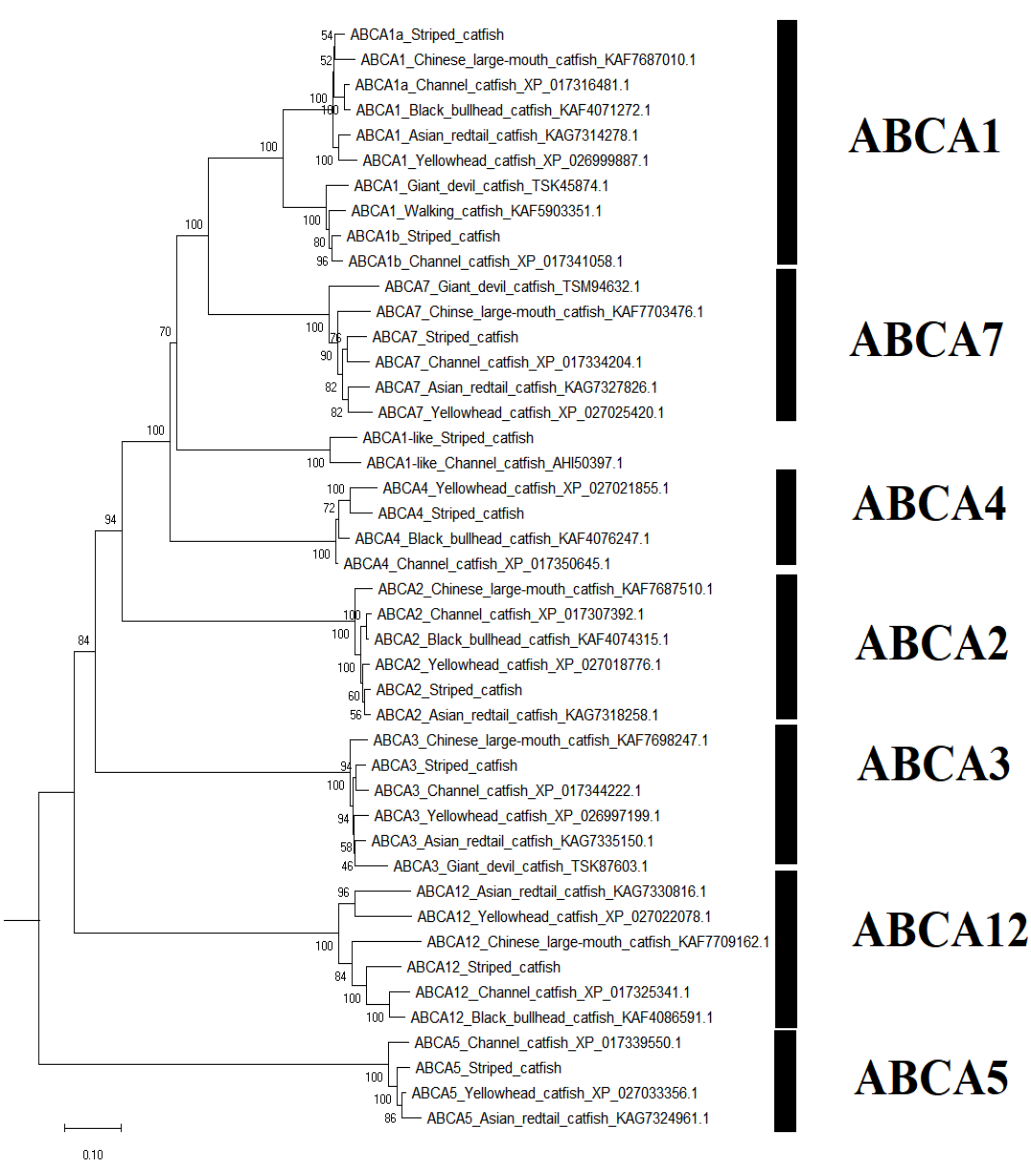
The maximum likelihood phylogenetic tree of ABCA subfamily, with 1000 bootstrap replications.

**Figure 2:**
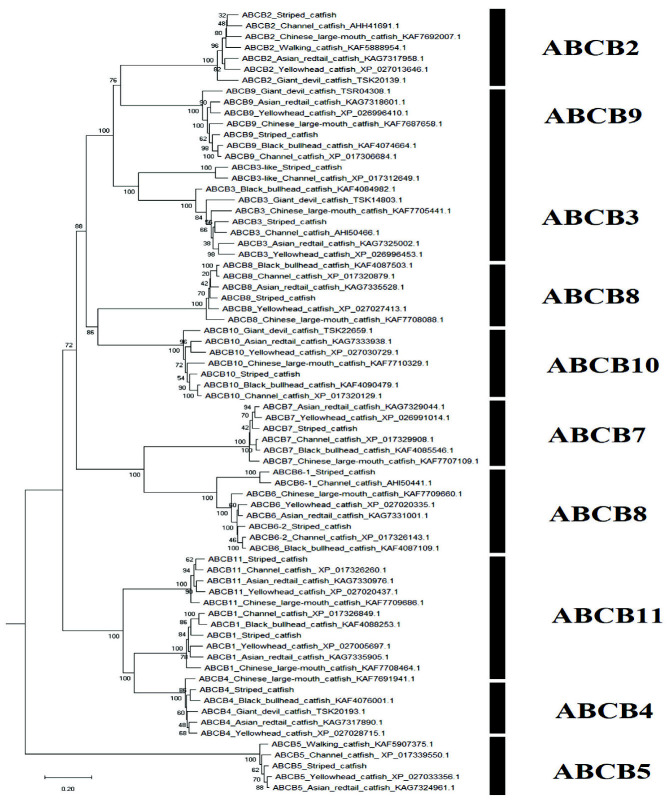
The maximum likelihood phylogenetic tree of ABCB subfamily, with 1000 bootstrap replications.

**Figure 3:**
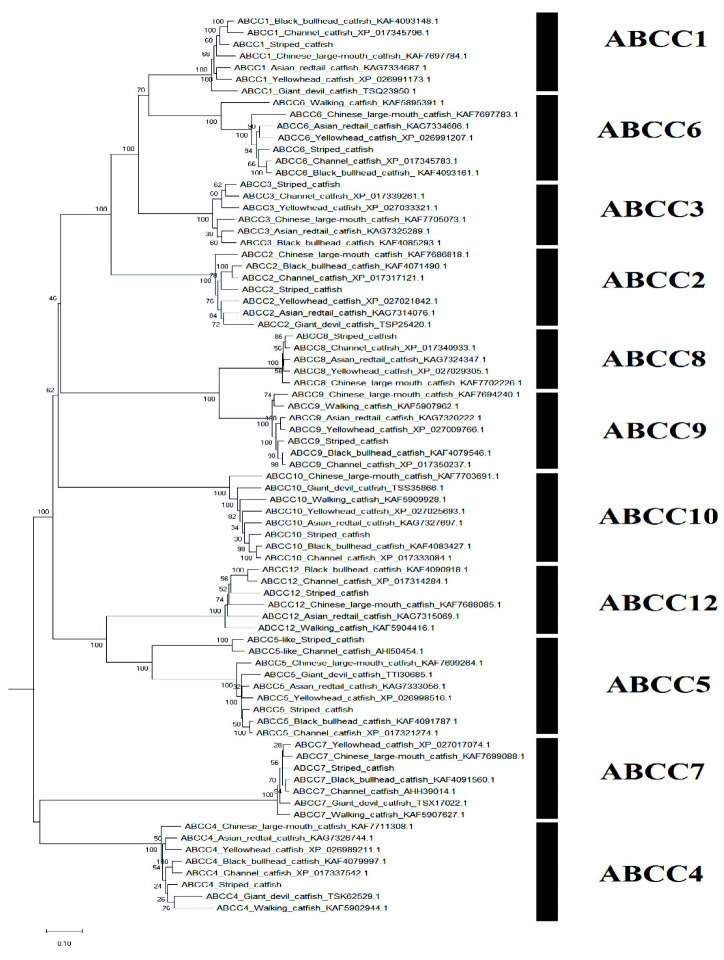
The maximum likelihood phylogenetic tree of ABCC subfamily, with 1000 bootstrap replications.

**Figure 4:**
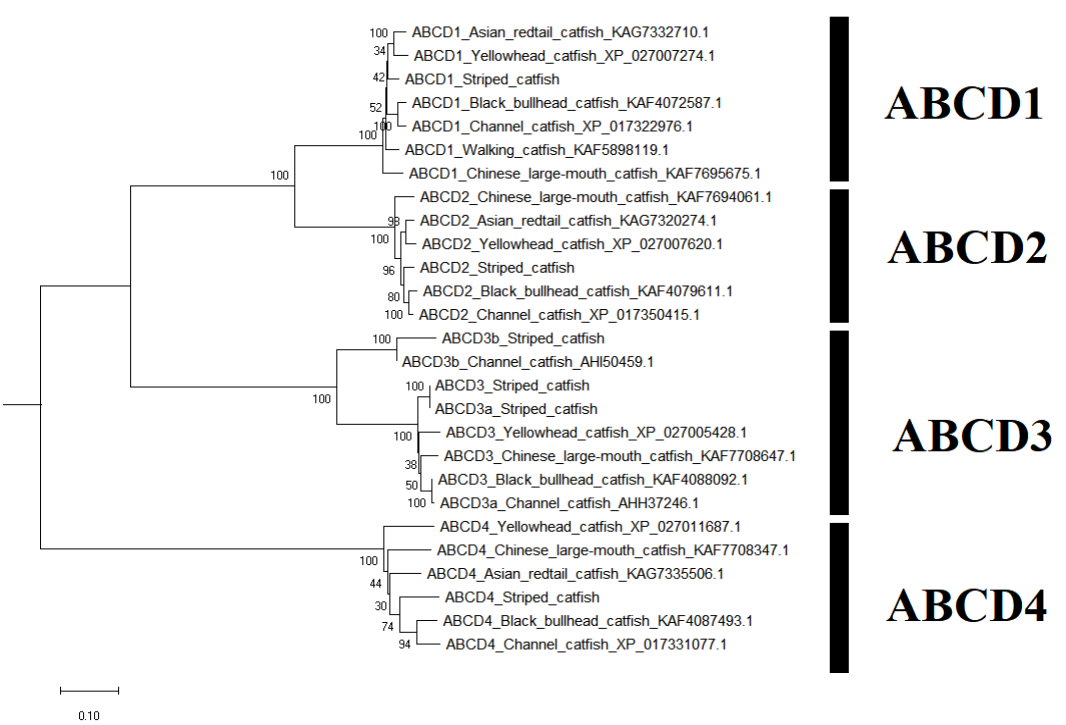
The maximum likelihood phylogenetic tree of ABCD subfamily, with 1000 bootstrap replications.

**Figure 5:**
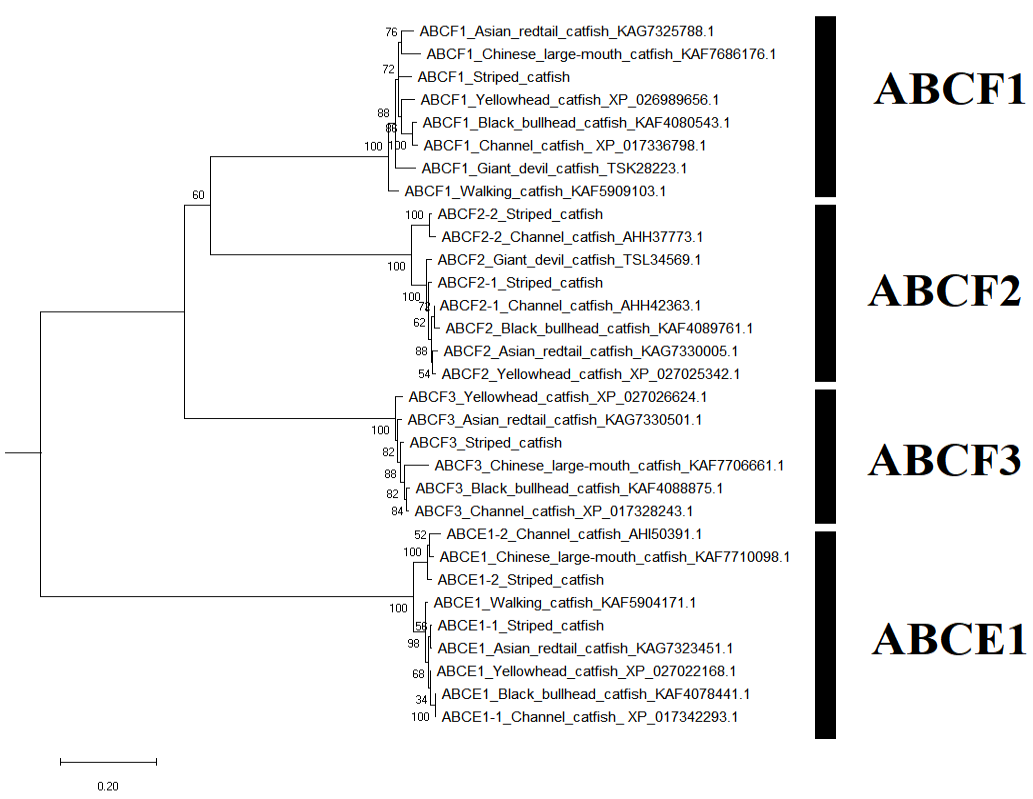
The maximum likelihood phylogenetic tree of ABCE-ABCF subfamily, with 1000 bootstrap replications.

**Figure 6:**
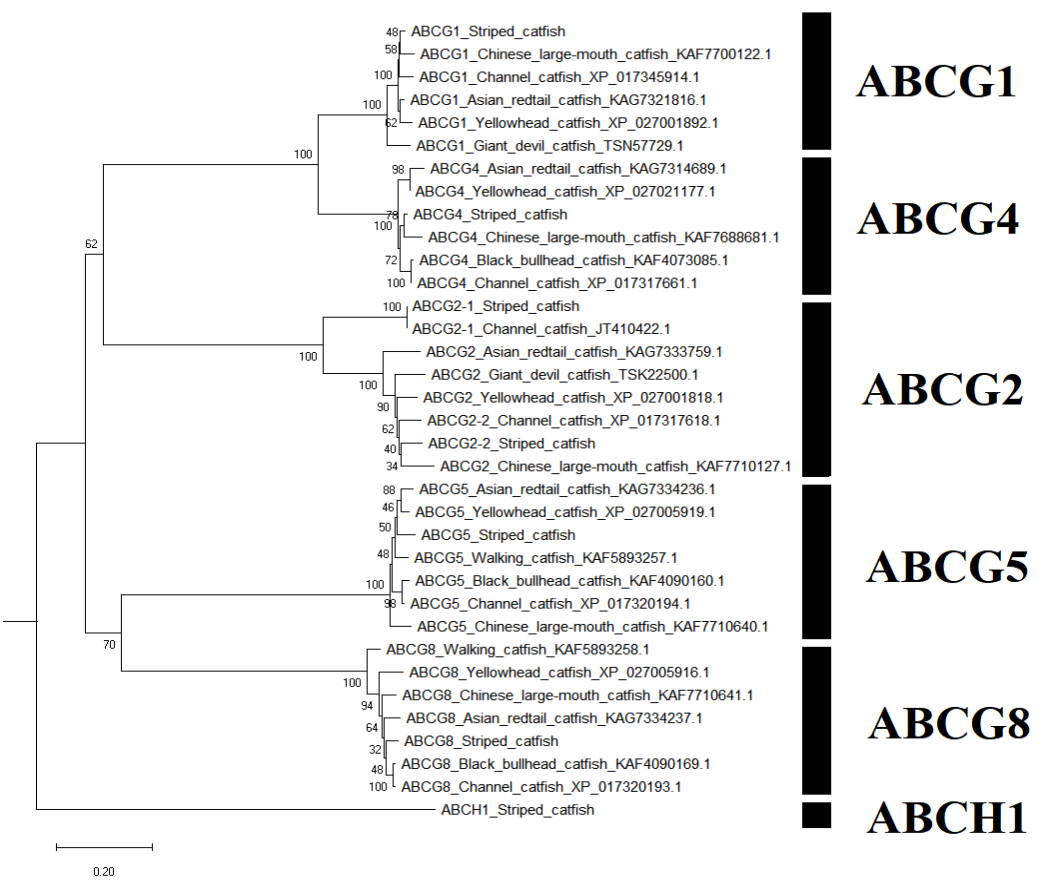
The maximum likelihood phylogenetic tree of ABCG-ABCH subfamily, with 1000 bootstrap replications.

**Figure 7:**
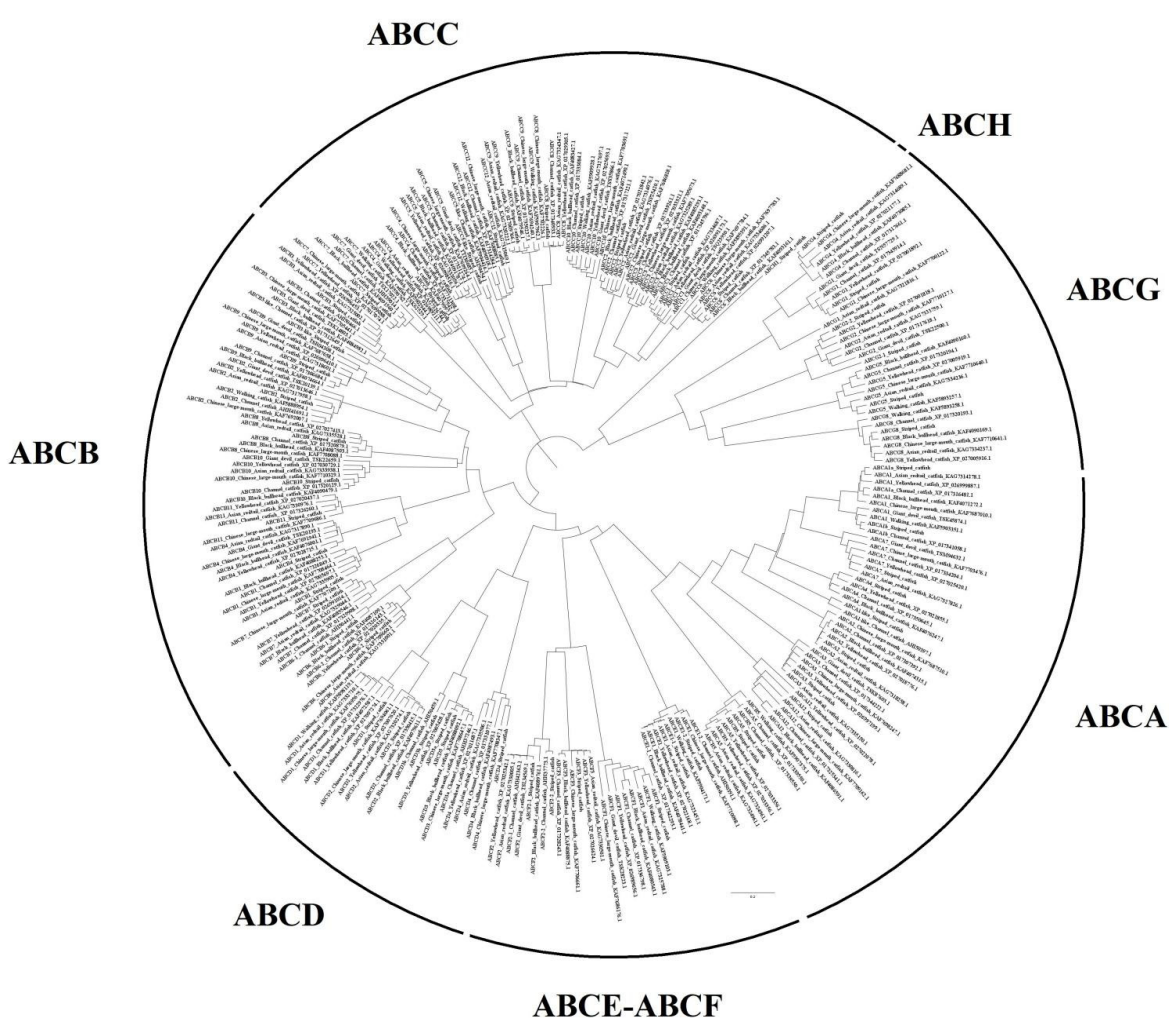
The maximum likelihood phylogenetic tree of all ABC proteins of catfishes, with 1000 bootstrap replications.

A sum of 52 ABC transporter genes were discovered in the striped catfish genome. Their amino acid lengths, molecular weights, subcellular localisations, theoretical isoelectric points as well as domain structures were summarised in Table 2. Domain structure was only compared against the channel catfish as this is the only catfish with full genome sequenced apart from the striped catfish while the ABC gene number within the genome was contrasted against several model organisms to provide an interspecies landscape view of ABC proteins evolution (Table 3). The identified 52 ABC transporters were divided into six subfamilies: 9 ABCAs, 13 ABCBs, 12 ABCCs, 5 ABCDs, 2 ABCEs, 4 ABCFs, 6 ABCGs and 1 ABCH. Motif (Figs. S1–S6 [see Appendices]) and phylogenetic analysis (Figs. 1–7) were conducted across all striped catfish ABC subfamilies.

### High Motif Conservation Observed Across the ABCA Subfamily Members

A total of nine ABCA genes were uncovered from the striped catfish genome, which encompasses ABCA1a, ABCA1b, ABCA-like, ABCA2, ABCA3, ABCA4, ABCA5, ABCA7 and ABCA12. All of them are full transporters and are located within the plasma membrane. The tra catfish ABCA12 is the largest protein of ABCA subfamily (338.61 kDa with 3026 aa) with the lowest pI of 5.25 (Table 2). The smallest *P. hypophthalmus* ABCA member is the ABCA3 (191.69 kDa with 1710 aa) but the highest pI value belongs to that of the ABCA1-like protein (7.52). The domain structures of tra catfish ABCA subfamily members mirrored that of the channel catfish with some exceptions and incomplete data impeding comparison, this holds true for ABCA1a, ABCA1b, ABCA1-like, ABCA2, ABCA5 and ABCA3. The coding sequences of channel catfish ABCA4, ABCA7 and ABCA12 are unavailable for comparison due to partial sequences deposited (Liu *et al*. 2013).

Looking at the motif distribution (Fig. S1: Appendix A), the ABCA3 and ABCA7 proteins are greatly conserved across the catfishes in terms of motif position, motif length and motif amount. Relatively high motif conservation pattern was also observed in ABCA1, ABCA2 and ABCA5 proteins across all catfishes. The motif distances of ABCA4 proteins varied significantly, especially that from black bullhead catfish and channel catfish. The spaces between the motifs within the ABCA12 proteins do not differ across all six catfishes. There is one missing motif (Motif 11, the retinal-specific rim ABC transporter [photoreceptor protein]) from the ABCA5 of black bullhead catfish. This motif is responsible in various pathways such as phosphotidylethanolamine flippase activity, phospholipid transporter activity, lipid transporter activity, ATP binding as well as ATPase-coupled intermembrane and transmembrane transporter activity (The UniProt Consortium 2021). However, the functional loss impacted by this motif loss is minor as there is high abundance of this motif within the protein.

The ABCA phylogenetic tree (Fig. 1) depicted that all ABCA proteins formed a cluster with their respective counterparts from other catfishes. The ABCA1a of channel catfish is more closely related to the ABCA1 of Asian redtail catfish instead of ABCA1a of the striped catfish. However, the ABCA1b proteins of both channel and striped catfish are grouped together with high bootstrap value of 96%. The ABCA1-like proteins of both striped and channel catfish are located far away from the ABCA1 cluster and are more closely associated with the ABCA7 group. A similar phenomenon has been previously observed by Liu *et al*. (2013) on channel catfish. They postulated that the ABCA7 proteins were possibly derived from ABCA1 by duplication event (Liu *et al*. 2013). Only the striped and channel catfishes contain three ABCA1 genes (Table 3) when interspecies ABC gene number were compared across a diverse range of well-documented model organisms. The ABCA6, ABCA8 and ABCA9 genes were found exclusively in mouse and human whereas the ABCA10 is exclusive to human only.

### ABCB4 is Present in Striped Catfish, Human, Mouse and Zebrafish, but Not in Channel Catfish

There are 13 ABCB family members identified from the striped catfish genome, namely ABCB1, ABCB2, ABCB3, ABCB3-like, ABCB4, ABCB5, ABCB6-1, ABCB6-2, ABCB7, ABCB8, ABCB9, ABCB10 and ABCB11. All ABCB transporters localise within the plasma membrane. The largest and smallest member of the ABCB subfamily of tra catfish are ABCB5 and ABCB6-2, respectively (Table 2). The highest and lowest recorded pI for the *P. hypophthalmus* ABCB subfamily are 10.15 (ABCB8) and 5.55 (ABCB9) correspondingly. The three full transporters of the tra catfish ABCB subfamily are the ABCB1, ABCB5 and ABCB11 proteins whereas the others are all half transporters. These transporters are conserved across channel and striped catfishes, except for ABCB6-1 where there are 11 TMDs in the striped catfish in contrast to the nine present in the channel catfish ABCB6-1 (Liu *et al*. 2013). The ABCB4 protein is absent in channel catfish (Liu *et al*. 2013) but present in mouse, human, zebrafish and tra catfish (Table 3).

Based on Fig. S2 (Appendix B), the motif distribution of the catfishes ABCB subfamily displayed a conserved pattern with some minor exceptions. For instance, Motif 11 (ABC Signature, Walker B and D-loop) and Motif 9 (P-loop containing nucleoside triphosphate hydrolases) are missing from the ABCB2 of the walking catfish and ABCB11 of Asian redtail catfish correspondingly. Besides, Motif 15 (Uncharacterised motif) is also absent in ABCB3 of the black bullhead catfish, ABCB5 of channel catfish and ABCB5 of walking catfish. Interestingly, the Motif 9 (P-loop containing nucleoside triphosphate hydrolases) of striped catfish ABCB5 was found at different location as compared to ABCB5 from other catfishes. This Motif 9 is responsible for various biological processes such as ATPase activity, ATP binding, hydrolase activity as well as zinc ion binding (The UniProt Consortium 2021).

The maximum likelihood phylogenetic tree of ABCB subfamily (Fig. 2) depicted distinctive clades separating the ABCB members accordingly. The ABCB3-like proteins of channel and tra catfish formed a strong clade of their own with 100% bootstrap value but they are still residing the same clade of ABCB3. Similarly, the ABCB6-1 proteins of striped and channel catfish obtained a bootstrap value of 100% for their cluster but that is not the case for ABCB6-2 proteins where the channel catfish ABCB6-2 is more closely related to the ABCB6 of black bullhead catfish than the ABCB6-2 of striped catfish. Liu *et al*. (2013) had previously reported a close synteny across ABCB1 and ABCB5 genes of channel catfish in comparison to that from Xenopus, chicken, mouse, human, zebrafish and medaka. The phylogenetic tree plotted by Liu *et al*. (2013) showed the ABCB1 and ABCB5 clades in close proximity, however in this study these two clades are sandwiching the ABCB4 clade. It is postulated that the lack of ABCB4 proteins from fish (only human and mouse ABCB4 used) included in the phylogenetic tree by Liu *et al*. (2013) has caused the weak separation between ABCB1 and ABCB5 proteins as the bootstrap values were low across these two clades in Liu *et al*. (2013).

### All Striped Catfish ABCC Subfamily Proteins Mirrored that of the Channel Catfish

The ABCC subfamily of striped catfish encompasses a sum of twelve ABCC members, including ABCC1, ABCC2, ABCC3, ABCC4, ABCC5, ABCC5-like, ABCC6, ABCC7, ABCC8, ABCC9, ABCC10 and ABCC12. All of them are full transporters and they are localised in the plasma membrane of the cell. The 1631 aa ABCC8 is the lengthiest member with a documented molecular weight of 182.48 kDa whereas the 1324 aa ABCC4 is the smallest of all with molecular weight of 149.15 kDa. The ABCC7 has the highest pI of 8.72 while the lowest pI belongs to that of ABCC9. All tra catfish ABCC domain structures echoed that of the channel catfish (Liu *et al*. 2013). The ABCC11 gene was only found in human whereas the ABCC13 protein is exclusive to zebrafish only (Table 3).

Referring to Fig. S3 (Appendix C), the motif distribution pattern is very conserved across all ABCC protein members of all catfishes, except for the ABCC1 of channel catfish and ABCC2 of giant devil catfish. Both Motif 2 (ABC Signature, Walker B and D-loop) and Motif 9 (H-loop/switch region) are missing from channel catfish ABCC1 as well as giant devil catfish ABCC2. Additionally, Motif 4 (Q-loop/lid) was also not found in the ABCC1 of channel catfish. This Q-loop, or the lid, is an indispensable element in couple drug binding to the ATP catalytic cycle (Zolnerciks *et al*. 2014). One notable observation is that the ABCC7 of striped catfish has Motif 9 (H-loop/switch region), which this motif is absent in all ABCC7 of other catfishes. H-loop, or the switch region, is obligated to catalyse ATP hydrolysis (Zhou *et al*. 2013).

The phylogenetic tree representing the ABCC subfamily (Fig. 3) illustrates the well-supported annotations of ABCC proteins. The multi-drug resistance proteins (ABCC1, ABCC2, ABCC3, ABCC6, ABCC9 and ABCC10) formed a monophyletic cluster whereas the rest formed another. The ABCC5-like proteins of striped catfish and channel catfish formed a strong cluster with bootstrap score of 100%. Yet, they are still attached to the major ABCC5 clade with bootstrap value of 100%. Generally, the ABCC subfamily tree reported in this study mirrored that of the tree constructed by Liu *et al*. (2013).

### Striped Catfish ABCD3a Did Not Form A Clade with ABCD3a of Channel Catfish

The ABCD subfamily of tra catfish consists of five protein members, namely ABCD1, ABCD2, ABCD3a, ABCD3b and ABCD4. All five members are half transporters and majority of them are localised in the plasma membrane, with the exception of ABCD2 where it is localised in the mitochondria. The ABCD1 can be found in both plasma membrane and mitochondria. The ABCD1 is the largest of all ABCDs and the ABCD3a is the smallest. The ABCD3a has the highest pI of 9.70 whereas the ABCD4 has the lowest value of 8.03. The domain structures of tra catfish ABCD proteins resemble closely to that of the channel catfish, with the ABCD4 as an minor exception. The ABCD4 of channel catfish contains three transmembrane domains whereas the striped catfish has two extra. All organisms examined in Table 3 contains at least one copy of each ABCD genes, except for tetradon with the lack of ABCD2 gene (Table 3).

At a glance on Fig. S4 (Appendix D), the motif arrangements of all ABCD1 and ABCD2 proteins portrayed high conservation. Only the walking catfish ABCD1 lacks a Motif 2 (Peroxysomal fatty acyl CoA transporter family protein). The ABCD3 proteins also showed relatively high resemblance to that of ABCD1 and ABCD2 proteins, except that they lack the two motifs flanking the both ends of ABCD1 and ABCD2 proteins. The ABCD4 proteins are quite distinctive from the other ABCD members in terms of motif arrangements, motif number and motif availability. Motifs such as Motif 9 (Peroxysomal fatty acyl CoA transporter family protein), Motif 10 (Peroxysomal fatty acyl CoA transporter family protein) and Motif 8 (Peroxysomal fatty acyl CoA transporter family protein) are absent from the ABCD proteins. The peroxysomal fatty acyl CoA transporter family protein functions as an assistant to the ABCD transporters to facilitate the cleaving of acyl CoA substrates before the reactivation by the peroxisomal synthetases (Baker *et al*. 2015).

The ABCD maximum likelihood phylogenetic tree (Fig. 4) showcases four major clades. The ABCD3b proteins of channel and tra catfish formed a strong clade with bootstrap score of 100%. Conversely, the channel catfish ABCD3a did not form a clade with ABCD3a of tra catfish, instead it formed a strong clade with the black bullhead catfish with 100% bootstrap value. The nomenclature of ABCD3b comes from both zebrafish and channel catfish where this duplicated ABCD3-like was named ABCD3b, however the orthology of this protein necessitates further verification.

### All ABCE-ABCF Subfamily Members are Found in All Organisms Examined in This Study

The ABCE-ABCF subfamily contains proteins with NBDs only and without TMDs. These proteins are termed non-functional transporters and they localise in cytoplasm and nucleus, unlike the majority of other ABC proteins which localise in the plasma membrane. Despite having the same amino acid length, both tra catfish ABCE1 have different molecular weights and theoretical isoletric points. The ABCF1 is the largest protein of the group and the ABCE1-2 topped the pI chart. All domain structures of tra catfish ABCE and ABCF proteins is the same as that of the channel catfish (Liu *et al*. 2013). All members of this subfamily are present in all organisms investigated in Table 3.

High motif conservation was observed across all ABCE and ABCF proteins (Fig. S5: Appendix E), with some exclusions. The Motif 5 (ABC transporter F family) of the striped catfish ABCE1-2 is located somewhere in the middle of protein instead of at the starting position. Both the channel catfish ABCE1-2 and Chinese large-mouth catfish ABCE1 has Motif 1 (ATPase components of ABC transporters with duplicated ATPase domains) located at the middle position instead of towards the end of protein. The ATPase components of ABC transporters with duplicated ATPase domains are involved in the DNA binding, ATP binding as well as ATPase-coupled transmembrane transporter activity (The UniProt Consortium 2021).

The phylogenetic tree of the ABCE-ABCF subfamily (Fig. 5) has well supported the annotations of all protein members. The channel catfish ABCE1-2, Chinese large-mouth catfish ABCE1 as well as striped catfish ABCE1-2 reside a slightly further clade from the major ABCE1 clade. The striped and channel catfish ABCF2-2 was grouped into a strong clade with 100% bootstrap score whereas the ABCF1-1 of the aforementioned catfishes are clustered closer with the other ABCF1 from other catfishes as compared to the ABCF2-2 proteins.

### ABCG3 is Mouse Exclusive While ABCH1 is Striped Catfish and Zebrafish Exclusive in this Study

Six ABCG proteins and one ABCH protein were identified from the striped catfish genome. All of them are half transporters, localising in the plasma membrane. All members of this subfamily do not differ much in molecular weights and amino acid lengths, with the highest recorded at 79.82 (ABCH1, 720 aa) and lowest recorded at 72.07 kDa (ABCG4, 643 aa). The highest pI was seen in ABCG5 protein (8.90) while the lowest pI was observed in ABCG8 protein. The members of this subfamily are unique from all other family members as their NBDs are located at the N-terminus instead of the C-terminus in other ABC proteins. Majority of the domain structures of tra catfish ABCG proteins closely resembles that of the channel catfish, except for ABCG1 and ABCG2-2 proteins. The reported ABCG1 proteins of the channel catfish was partial, therefore comparison cannot be done. The tra catfish ABCG2-2 protein has two less TMDs as compared to that of the channel catfish. The ABCG3 gene is absent in all organisms examined in Table 3, with the mouse as an exclusion. The ABCH1 gene is only found in striped catfish and zebrafish.

Looking at Fig. S6 (Appendix F), the motif distribution pattern is highly identical across ABCG1 and ABCG2 proteins. The ABCG4 proteins have an extra Motif 3 (Eye pigment precursor transporter family protein) at the N-terminus. Interestingly, the Motif 15 (Uncharacterised motif) of the ABCG5 proteins are located at the N-terminus instead of the C-terminus as observed in ABCG1, ABCG2, ABCG8 and ABCG4 proteins. The ABCG8 proteins have Motif 13 (Uncharacterised motif) in place of that N-terminus Motif 15 found in the ABCG5 proteins. The ABCH1 only contains Motif 3 (Eye pigment precursor transporter family protein) and Motif 1 (Walker B and D-loop). The eye pigment precursor transporter family protein serves as an orchestrator for pigment binding, ATP binding as well as ATPase-coupled transmembrane transporter activity (The UniProt Consortium 2021). The maximum likelihood phylogenetic tree of the ABCG-ABCH subfamily (Fig. 6) has also supported the annotations of all protein members of this subfamily. The ABCG2-1 proteins of channel and striped catfish formed a 100% bootstrap scored clade under the major ABCG2 subclade.

### Eight Striped Catfish ABC Transporters are Duplicated and Twelve Genes are Lost

Teleost fishes are known to have undergone genome duplication to have two paralogous copies of a myriad of genes in contrast to the only one ortholog as seen in tetrapods (Hoegg *et al*. 2004). Gene duplication as a result of evolution has resulted in the emergence of new genes equipped with neofunctions or partitioned functions (Liu *et al*. 2013).

There are a number of ABC transporters in striped catfish that have been identified to have experienced gene duplications. These ABC transporter genes include ABCA1, ABCB3, ABCB6, ABCC5, ABCD3, ABCE1, ABCF2 as well as ABCG2 (Table 2). These genes are postulated to be closely associated with the fish-specific genome duplication events (Liu *et al*. 2013). On the other hand, the single copy genes found within the tra and channel catfish genomes, namely ABCA4, ABCC4, ABCC6 and ABCG4, were believed to have lost subsequent to the whole genome duplication of these teleost fishes (Liu *et al*. 2013). Interestingly, some genes such as ABCA6, ABCA8, ABCA9, ABCA10, ABCA12, ABCA13, ABCA14, ABCA15, ABCA17, ABCB4, ABCC11 as well as ABCG3, are all absent from all the fish genomes examined in Table 3. These non-fish ABC transporter genes were deemed to have lost during the genome duplication events that diverge tetrapods from teloest fishes (Dean & Annilo 2005; Annilo *et al*. 2006) The ABCA6, ABCA8, ABCA9, ABCA10, ABCA12 as well as ABCA13 are postulated to be mammal-specific.

The striped catfish contains all the ABC transporters identified in the channel catfish genome and an additional of two ABC proteins not found in the channel catfish, namely the ABCB4 and ABCH1 proteins. The ABCB4 was found in human, mouse, striped catfish and zebrafish, but absent in channel catfish. This protein is a liver-specific transporter of phosphatidycholine (a mammalian bile compound) (Annilo *et al*. 2006). The lack of this protein suggested the elimination of the need for phospholipids in some teloest fishes (Annilo *et al*. 2006). These phospholipids are essential parts of the pulmonary surfactant utilised to lower the surface tension of the respiratory organ (Daniels *et al*. 1998). Hence, it is postulated that teleost fishes that are capable of air-breathing like the zebrafish and striped catfish may necessitate the ABCB4 protein for oxygen harvest from the air whereby it is needed to lubricate the respiratory tracts subsequent to dry air inhalation. The ABCH1 was only found in zebrafish and striped catfish among all the vertebrate genomes investigated in Table 3. In fact, this protein is also absent in yeast, plants, mammals and worms, but it is present in all insects, non-insect protozoans, Sarawak rasbora fish, non-insect metazoans, green spotted pufferfish, sea urchin as well as non-insect arthropods (Guo *et al*. 2015b; Jeong *et al*. 2015; Lim *et al*. 2018). In zebrafish, this protein was expressed in the gills, brain, kidney and intestine (Popovic *et al*. 2010). Popovic *et al*. (2010) postulated that the ABCH1 protein may have similar multixenobiotic defence role as the ABCG2 protein. Looking at all the fishes that contain the ABCH1 gene, one major similarity is that all of them are air-breather, hence it is postulated that this ABCH1 protein may be required for the detoxification of the inhaled air. Nonetheless, further investigation is needed to functionally characterise this protein in striped catfish in order to support the aforementioned postulations.

### Orthology and Potential Functional Inferences of Striped Catfish ABC Transporters

The ABC transporters have been intensively studied in human genetics where the majority of them are actively involved in pivotal biological processes and the onset of genetic diseases (Dean & Annilo 2005). These orthologs may provide clues towards the duplication of some ABC genes in teleosts in order to assist them to adapt to the aquatic environments.

In vertebrates, the ABCA1 proteins are the main cholesterol transporters. They are actively involved in the flopping of the inner leaflet cholesterol to the outer leaflet of the plasma membrane (Ogasawara *et al*. 2020). These proteins orchestrates and maintains a low cholesterol level in the inner leaflet of the plasma membrane, this makes the lipid a membranous signal molecule (Ogasawara *et al*. 2020). Ogasawara *et al*. (2020) deemed that the ABCA1 is the major accelerator of vertebrate evolution. The ABCA4 proteins are the photoreceptor rim proteins that are expressed exclusively in the retina (Sun & Nathans 2000). This protein plays multiple roles in early vertebrate development and one of the major function is the governance of retina-specific pathways in mammals like rodent, human and mouse (Borst & Elferink 2002). The duplication of ABCA1 and ABCA4 genes in most of the fish genomes in Table 3 signals dire need for further investigation on the association of high metabolic energy consumption of aquatic fishes and cholesterol translocation speed as well as whether retina cell activities are required twice as much in fishes than in mammals.

The ABCB3 proteins are antigen peptide transporters present in zebrafish, tra catfish and channel catfish, but absent in species that evolved earlier like the lampreys (Ren *et al*. 2015). These proteins are closely associated with the gnathostomes adaptive immune system as they translocate peptides to the endoplasmic reticulum to aid in the formation of class I major histocompatibility complex molecules (Michalova *et al*. 2000). The zebrafish ABCB3 and ABCB7 were proven as efficient metal detoxicants not so long ago (Lerebours *et al*. 2016). The ABCB6 proteins participate in the iron metabolism and Fe/S protein precursors translocation (Zutz *et al*. 2009). The expression of this protein was found high in muscles and intestines during early metamorphic stages of lampreys (Ren *et al*. 2015). Therefore, it is postulated that the ABCB3 and ABCB6 gene duplication in channel and striped catfishes may be essential for the high detoxification needs in fish as compared to mammals (Liu *et al*. 2013).

Majority of the ABCC members are involved in toxic translocation out of the cells. The ABCC5 proteins are highly expressed in glia, blood-brain barrier and neurons (Jansen *et al*. 2015). They are indispensable efflux transporters of glutamate conjugates and analogs, governing the activity of the excitatory neurotransmitters in the brain (Jansen *et al*. 2015). Besides, this protein also orchestrates the depth of the anterior chamber and is directly linked toward the primary angle closure glaucoma (Nongpiur *et al*. 2014). Human cervical cancer cells expressing moderate ABCC5 protein levels were discovered to experience exponential growth (Eggen *et al*. 2012). In fishes, this protein is highly expressed in the muscles, brain, gills and digestive tracts of the zebrafish (Bolbol *et al*. 2013). The elevated expression of ABCC5 genes was induced with the presence of *Tubifex* worms and metallic (copper and cadmium) contamination (Bolbol *et al*. 2013). The duplication of this gene in catfishes, tilapia and medaka may have similar implications or a more substantial role in assisting these fishes in their adaptations to chemically harsh environments.

Each of the ABCD subfamily members has diverse functions unlike proteins from other ABC subfamilies. The ABCD1 and ABCD2 proteins translocate long and very long chain fatty acids into peroxisomes, while the ABCD4 transfers lysosomal vitamin B12 to the cytosol (Kawaguchi & Morita 2016). On the other hand, the ABCD3 is responsible for the translocation of branched chain acyl-CoA towards the peroxisomes (Kawaguchi & Morita 2016). The price of having defective ABCD1 and ABCD3 is one of the most lethal as this can lead to neurodegenerative disease and hepatosplenomegaly liver disease (Mosser *et al*. 1993; Ferdinandusse *et al*. 2015). The duplication of ABCD3 gene was observed in zebrafish as well as both the catfishes, therefore it is imperative to decipher its associated metabolic pathways, especially when both catfishes are highly demanded important sources of great quality unsaturated fatty acids.

The expression of ABCE1, ABCF1 and ABCF2 was seen in all tissues in lampreys, Sarawak rasbora and zebrafish (Ren *et al*. 2015; Lim *et al*. 2018; Newman *et al*. 2019). The ABCE1 protein has diverse roles such as ribosome recycling, translation initiation, termination and elongation (Chen *et al*. 2006; Barthelme *et al*. 2011; Pisareva *et al*. 2011). The efficacy of the ABCF2 protein in resisting cisplatin-induced apoptosis in ovarian cancer cells has been documented by Bao *et al*. (2018). Both ABCE1 and ABCF2 genes are duplicated in channel and striped catfishes, while there are three ABCE1 in cod and two ABCF2 in zebrafish, these extra copies would serve as a replacement in times when one of them is defective in nature, mutated or halted of their housekeeping obligations.

The ABCG2 protein is one of the most well-studied members of the ABCG subfamily. This protein is also named mitoxantrone-resistance protein, breast cancer resistance associated protein and placenta-specific ABC proteins for the diverse pathways it is involved in (Dermauw & van Leeuwen 2014; Ferreira *et al*. 2014). Furthermore, it also facilitates the restricted absorption of pharmaceutics into the intestine, exports urate from the kidney as well as provides shield for haematopoietic stem cells against heme-induced toxins (Kerr *et al*. 2011; Vlaming *et al*. 2009; Woodward *et al*. 2011). In fishes, the rainbow trout ABCG2a was proven an effective guard against environmental toxins (Zaja *et al*. 2016). The research on ABCG2 genes of economically valuable fishes like the catfishes is extremely essential to minimise the harvest loss due to the presence of environmentally induced toxins.

## CONCLUSION

A sum of 52 ABC transporters were unearthed from the striped catfish genome. The motif analysis has revealed several exclusive characteristics of some catfishes in this study. The phylogenetic analysis has proven its efficacy in the successful annotations of these transporter proteins. Duplicated genes such as ABCA1, ABCB3, ABCB6, ABCC5, ABCD3, ABCE1, ABCF2 as well as ABCG2 were found within the striped and channel catfish genomes. The objectives set for this study have been achieved, leaving some windows for more knowledge gaps to be filled soon. This whole set of ABC transporters channels valuable genomic landscape for future ecotoxicological, biochemical and physiological researches in striped catfish.

## Figures and Tables

**Table 1:** The transcriptome sequencing data.

Number of contigs (>= 0 bp)	154,159
Number of contigs (>= 1000 bp)	48,049
Number of contigs (>= 5000 bp)	3,634
Number of contigs (>= 10,000 bp)	126
Number of contigs (>= 25,000 bp)	0
Number of contigs (>= 50,000 bp)	0
Total length (>= 0 bp)	168,216,156
Total length (>= 1000 bp)	125,526,508
Total length (>= 5000 bp)	23,574,416
Total length (>= 10,000 bp)	1,464,951
Total length (>= 25,000 bp)	0
Total length (>= 50,000 bp)	0
Total number of contigs (>= 500 bp)	73,879
Largest contig	17,647
Total length	143,584,872
GC (%)	44.52
N50	2,729
N75	1,594
L50	17,010
L75	34,065
Number of Ns per 100 kbp	0.00

**Table 2:** The protein profiles of the 52 striped catfish ABC proteins.

Gene/Protein	Amino acid length (aa)	Molecular weight (kDa)	Theoretical isoelectric point (pI)	Subcellular localisation	Domain structure
*ABCA1a*	2269	254.54	6.24	Plasma membrane	(6/7TMD-NBD)2
*ABCA1b*	2269	254.27	6.07	Plasma membrane	(6/7TMD-NBD)2
*ABCA1-like*	2287	255.56	7.52	Plasma membrane	(7TMD-NBD)2
*ABCA2*	2502	279.74	6.48	Plasma membrane	(8/5TMD-NBD)2
*ABCA3*	1710	191.69	6.37	Plasma membrane	(7TMD-NBD)2
*ABCA4*	2341	264.94	6.46	Plasma membrane	(5/6TMD-NBD)2
*ABCA5*	1653	186.09	6.96	Plasma membrane	(7TMD-NBD)2
*ABCA7*	2328	260.86	6.38	Plasma membrane	(5/6TMD-NBD)2
*ABCA12*	3026	338.61	5.25	Plasma membrane	(5/6TMD-NBD)2
*ABCB1*	1337	147.32	8.92	Plasma membrane	(5/6TMD-NBD)2
*ABCB2*	726	81.27	7.63	Plasma membrane	7TMD-NBD
*ABCB3*	717	80.93	9.55	Plasma membrane	7TMD-NBD
*ABCB3-like*	719	80.86	8.53	Plasma membrane	5TMD-NBD
*ABCB4*	1277	141.08	8.56	Plasma membrane	(6/5TMD-NBD)2
*ABCB5*	1653	186.09	6.96	Plasma membrane	(7TMD-NBD)2
*ABCB6-1*	852	96.25	8.14	Plasma membrane	11TMD-NBD
*ABCB6-2*	650	73.61	7.28	Plasma membrane	6TMD-NBD
*ABCB7*	744	81.99	9.47	Plasma membrane	5TMD-NBD
*ABCB8*	709	76.77	10.15	Plasma membrane	5TMD-NBD
*ABCB9*	787	87.07	5.55	Plasma membrane	7TMD-NBD
*ABCB10*	704	77.04	9.34	Plasma membrane	5TMD-NBD
*ABCB11*	1324	146.18	7.22	Plasma membrane	5TMD-NBD
*ABCC1*	1515	169.65	7.75	Plasma membrane	5TMD-(6/5TMD-NBD)2
*ABCC2*	1565	175.49	8.62	Plasma membrane	5TMD-(6/5TMD-NBD)2
*ABCC3*	1538	173.74	6.25	Plasma membrane	5TMD-(4/5TMD-NBD)2
*ABCC4*	1324	149.15	8.16	Plasma membrane	(7/5TMD-NBD)2
*ABCC5*	1423	158.27	8.23	Plasma membrane	(6/5TMD-NBD)2
*ABCC5-like*	1398	155.51	8.37	Plasma membrane	(5/4TMD-NBD)2
*ABCC6*	1506	169.54	6.76	Plasma membrane	5TMD-(5/2TMD-NBD)2
*ABCC7*	1480	168.51	8.72	Plasma membrane	(4/6TMD-NBD)2
*ABCC8*	1631	182.48	7.11	Plasma membrane	5TMD-(6/4TMD-NBD)2
*ABCC9*	1595	180.19	6.23	Plasma membrane	5TMD-(6/5TMD-NBD)2
*ABCC10*	1547	171.98	6.74	Plasma membrane	5TMD-(6/5TMD-NBD)2
*ABCC12*	1410	158.07	7.86	Plasma membrane	(6/5TMD-NBD)2
*ABCD1*	780	88.53	8.79	Plasma membrane and mitochondria	NBD
*ABCD2*	746	83.10	9.17	Mitochondria	NBD
*ABCD3a*	658	73.58	9.70	Plasma membrane	4TMD-NBD
*ABCD3b*	665	75.66	9.39	Plasma membrane	4TMD-NBD
*ABCD4*	603	68.65	8.03	Plasma membrane	5TMD-NBD
*ABCE1-1*	599	67.35	8.30	Cytoplasm and nucleus	NBD-NBD
*ABCE1-2*	599	67.60	8.46	Cytoplasm and nucleus	NBD-NBD
*ABCF1*	864	97.93	5.98	Nucleus	NBD-NBD
*ABCF2-1*	609	69.85	6.90	Cytoplasm and nucleus	NBD-NBD
*ABCF2-2*	609	69.66	6.93	Cytoplasm and nucleus	NBD-NBD
*ABCF3*	710	80.07	5.33	Cytoplasm	NBD-NBD
*ABCG1*	672	74.96	6.89	Plasma membrane	NBD-7TMD
*ABCG2-1*	643	72.15	8.76	Plasma membrane	NBD-5TMD
*ABCG2-2*	656	73.41	8.81	Plasma membrane	NBD-5TMD
*ABCG4*	643	72.07	7.85	Plasma membrane	NBD-7TMD
*ABCG5*	655	73.61	8.90	Plasma membrane	NBD-6TMD
*ABCG8*	679	76.82	6.71	Plasma membrane	NBD-5TMD
*ABCH1*	720	79.52	8.51	Plasma membrane	NBD-6TMD

**Table 3:** The ABC gene number comparison across various vertebrates.

Gene	Striped catfish	Channel catfish	Zebrafish	Medaka	Fugu	Tetradon	Stickleback	Tilapia	Cod	Mouse	Coelacanth	Human
*ABCA1*	3	3	2	2	2	2	2	2	2	1	1	1
*ABCA2*	1	1	1	0	0	0	0	0	0	1	0	1
*ABCA3*	1	1	1	1	1	1	1	1	1	1	1	1
*ABCA4*	1	1	2	2	2	1	2	2	2	1	1	1
*ABCA5*	1	1	1	0	1	1	1	1	1	1	1	1
*ABCA6*	0	0	0	0	0	0	0	0	0	1	0	1
*ABCA7*	1	1	1	1	1	1	1	0	1	1	1	1
*ABCA8*	0	0	0	0	0	0	0	0	0	2	0	1
*ABCA9*	0	0	0	0	0	0	0	0	0	1	0	1
*ABCA10*	0	0	0	0	0	0	0	0	0	0	0	1
*ABCA12*	1	1	1	1	1	0	1	1	1	1	1	1
*ABCA13*	0	0	0	0	0	0	0	0	0	1	0	1
*ABCA14*	0	0	0	0	0	0	0	0	0	1	0	0
*ABCA15*	0	0	0	0	0	0	0	0	0	1	0	0
*ABCA17*	0	0	0	0	0	0	0	0	0	1	0	0
*ABCB1*	1	1	1	1	0	0	1	1	1	2	0	1
*ABCB2*	1	1	1	1	1	1	1	1	1	1	1	1
*ABCB3*	2	2	1	0	0	0	0	0	0	1	0	1
*ABCB4*	1	0	0	0	0	0	0	0	0	1	0	1
*ABCB5*	1	1	1	0	0	0	0	0	0	1	1	1
*ABCB6*	2	2	2	1	2	2	2	2	1	1	1	1
*ABCB7*	1	1	1	1	1	1	1	1	1	1	1	1
*ABCB8*	1	1	1	1	1	1	1	1	1	1	1	1
*ABCB9*	1	1	1	1	1	1	1	0	1	1	1	1
*ABCB10*	1	1	1	1	1	1	1	1	1	1	1	1
*ABCB11*	1	1	2	1	1	1	1	0	1	1	1	1
*ABCC1*	1	1	1	1	1	1	1	1	0	1	1	1
*ABCC2*	1	1	1	1	1	1	1	1	1	1	1	1
*ABCC3*	1	1	1	1	1	1	1	1	0	1	0	1
*ABCC4*	1	1	1	2	3	4	4	3	3	1	1	1
*ABCC5*	2	2	1	2	1	1	1	2	1	1	0	1
*ABCC6*	1	1	3	1	1	2	2	1	1	1	1	1
*ABCC7*	1	1	1	0	0	0	0	0	1	1	0	1
*ABCC8*	1	1	3	1	1	1	1	1	1	1	1	1
*ABCC9*	1	1	1	0	0	0	0	0	0	1	1	1
*ABCC10*	1	1	1	0	1	1	1	1	1	1	1	1
*ABCC11*	0	0	0	0	0	0	0	0	0	0	0	1
*ABCC12*	1	1	1	0	1	1	1	0	1	1	0	1
*ABCC13*	0	0	1	0	0	0	0	0	0	0	0	0
*ABCD1*	1	1	1	1	1	1	1	1	0	1	1	1
*ABCD2*	1	1	2	1	1	0	1	1	1	1	1	1
*ABCD3*	2	2	2	1	1	1	1	1	1	1	1	1
*ABCD4*	1	1	1	1	1	1	1	1	1	1	1	1
*ABCE1*	2	2	1	1	1	1	1	1	3	1	1	1
*ABCF1*	1	1	1	1	1	3	1	1	1	1	1	1
*ABCF2*	2	2	2	1	1	1	1	1	1	1	1	1
*ABCF3*	1	1	1	1	1	1	1	1	1	1	1	1
*ABCG1*	1	1	1	1	1	0	0	1	0	1	1	1
*ABCG2*	2	2	3	2	2	2	2	2	2	1	0	1
*ABCG3*	0	0	0	0	0	0	0	0	0	1	0	0
*ABCG4*	1	1	2	2	3	2	2	2	1	1	1	1
*ABCG5*	1	1	1	1	1	1	1	1	1	1	1	1
*ABCG8*	1	1	1	1	1	1	1	1	1	1	1	1
*ABCH1*	1	0	1	0	0	0	0	0	0	0	0	0

## APPENDIX

### Appendix A

Figure S1 The ABCA subfamily motif analysis.

### APPENDIX B

Figure S2 The ABCB subfamily motif analysis.

### APPENDIX C

Figure S3 The ABCC subfamily motif analysis.

### APPENDIX D

Figure S4 The ABCD subfamily motif analysis.

### APPENDIX E

Figure S5 The ABCE-ABCF subfamily motif analysis.

### APPENDIX F

Figure S6 The ABCG-ABCH subfamily motif analysis.

## References

[b1-tlsr-33-2-257] Andersen V, Vogel LK, Kopp TI, Sæbø M, Nonboe AW, Hamfjord J, Kure EH, Vogel U (2015). High ABCC2 and low ABCG2 gene expression are early events in the colorectal adenoma-carcinoma sequence. PLoS ONE.

[b2-tlsr-33-2-257] Annilo T, Chen ZQ, Shulenin S, Costantino J, Thomas L, Lou H, Stefanov S, Dean M (2006). Evolution of the vertebrate ABC gene family: Analysis of gene birth and death. Genomics.

[b3-tlsr-33-2-257] Bailey TL, Johnson J, Grant CE, Noble WS (2015). The MEME suite. Nucleic Acids Research.

[b4-tlsr-33-2-257] Baker A, Carrier DJ, Schaedler T, Waterham HR, van Roermund CW, Theodoulou FL (2015). Peroxisomal ABC transporters: Functions and mechanism. Biochemical Society Transactions.

[b5-tlsr-33-2-257] Barthelme D, Dinkelaker S, Albers SV, Londei P, Ermler U, Tampé R (2011). Ribosome recycling depends on a mechanistic link between the FeS cluster domain and a conformational switch of the twin-ATPase ABCE1. PNAS.

[b6-tlsr-33-2-257] Bao L, Wu J, Dodson M, de la Vega EM, Ning Y, Zhang Z, Yao M, Zheng DD, Xu C, Yi X (2018). *ABCF2*, an Nrf2 target gene, contributes to cisplatin resistance in ovarian cancer cells. Molecular Carcinogenesis.

[b7-tlsr-33-2-257] Bolbol DMS, Rabie T, Ahmed AI, Zakaria S, Bourdineaud JP (2013). Zebrafish ABCC5 gene expression in relation to metallic contamination and presence of Tubifex worms. Egyptian Journal of Aquatic Biology and Fisheries.

[b8-tlsr-33-2-257] Borst P, Elferink RO (2002). Mammalian ABC transporters in health and disease. Annual Review of Biochemistry.

[b9-tlsr-33-2-257] Buchfink B, Xie C, Huson DH (2015). Fast and sensitive protein alignment using DIAMOND. Nature Methods.

[b10-tlsr-33-2-257] Chen ZQ, Dong J, Ishimura A, Daar I, Hinnebusch AG, Dean M (2006). The essential vertebrate ABCE1 protein interacts with eukaryotic initiation factors. Journal of Biological Chemistry.

[b11-tlsr-33-2-257] Chung HH, Kamar CKA, Lim LWK, Liao Y, Lam TT, Chong YL (2020). Sequencing and characterisation of complete mitogenome DNA for *Rasbora hobelmani* (Cyprinidae) with phylogenetic consideration. Journal of Ichthyology.

[b12-tlsr-33-2-257] Daniels CB, Orgeig S, Wood PG, Sullivan LC, Lopatko OV, Smits AW (1998). The changing state of surfactant lipids: New insights from ancient animals. American Zoologist.

[b13-tlsr-33-2-257] De Castro E, Sigrist CJA, Gattiker A, Bulliard V, Langendijk, -Genevaux PS, Gasteiger E, Bairoch A, Hulo N (2006). ScanProsite: Detection of PROSITE signature matches and ProRule-associated functional and structural residues in proteins. Nucleic Acids Research.

[b14-tlsr-33-2-257] Dean M, Annilo T (2005). Evolution of the ATP-binding cassette (ABC) transporter superfamily in vertebrates. Annual Review of Genomics and Human Genetics.

[b15-tlsr-33-2-257] Dermauw W, Van Leeuwen T (2014). The ABC gene family in arthropods: Comparative genomics and role in insecticide transport and resistance. Insect Biochemistry and Molecular Biology.

[b16-tlsr-33-2-257] Eggen T, Sager G, Berg T, Nergaard B, Moe BTG, Ørbo A (2012). Increased gene expression of the ABCC5 transporter without distinct changes in the expression of PDE5 in human cervical cancer cells during growth. Anticancer Research.

[b17-tlsr-33-2-257] Ferdinandusse S, Jimenez-Sanchez G, Koster J, Denis S, Van Roermund CW, Silva-Zolezzi I, Moser AB, Visser WF, Gulluoglu M (2015). A novel bile acid biosynthesis defect due to a deficiency of peroxisomal ABCD3. Human Molecular Genetics.

[b18-tlsr-33-2-257] Ferreira M, Costa J, Reis-Henriques MA (2014). ABC transporters in fish species: A review. Frontiers in Physiology.

[b19-tlsr-33-2-257] Gao Z, You X, Zhang X, Chen J, Xu T, Huang Y, Lin X, Xu J, Bian C, Shi Q (2021). A chromosome-level genome assembly of the striped catfish (*Pangasianodon hypophthalmus*). Genomics.

[b20-tlsr-33-2-257] Guo F, Ding Y, Caberoy N, Alvarado G, Wang F, Chen R, Li W (2015a). ABCF1 extrinsically regulates retinal pigment epithelial cell phagocytosis. Molecular Biology of the Cell.

[b21-tlsr-33-2-257] Guo Z, Kang S, Zhu X, Xia J, Wu Q, Wang S, Xie W, Zhang Y (2015b). The novel ABC transporter ABCH1 is a potential target for RNAi-based insect pest control and resistance management. Scientific Reports.

[b22-tlsr-33-2-257] Gurevich A, Saceliev V, Vyahhi N, Tesler G (2013). QUAST: Quality assessment tool for genome assemblies. Bioinformatics.

[b23-tlsr-33-2-257] Hoegg S, Brinkmann H, Taylor JS, Meyer A (2004). Phylogenetic timing of the fish-specific genome duplication correlates with the diversification of teleost fish. Journal of Molecular Evolution.

[b24-tlsr-33-2-257] Jansen RS, Mahakena S, de Haas M, Borst P, van de Watering K (2015). ATP-binding cassette subfamily C member 5 (ABCC5) functions as an efflux transporter of glutamate conjugates and analogs. Journal of Biological Chemistry.

[b25-tlsr-33-2-257] Jeong CB, Kim BM, Kang HM, Choi IK, Rhee JS, Lee JS (2015). Marine medaka ATP-binding cassette (ABC) superfamily and new insight into teleost abch nomenclature. Scientific Reports.

[b26-tlsr-33-2-257] Kawaguchi K, Morita M (2016). ABC transporter subfamily D: Distinct differences in behavior between ABCD1–3 and ABCD4 in subcellular localization, function, and human disease. BioMed Research International.

[b27-tlsr-33-2-257] Kerr ID, Haider AJ, Gelissen IC (2011). The ABCG family of membrane associated transporters: you don’t have to be big to be mighty. British Journal of Pharmacology.

[b28-tlsr-33-2-257] Kim OTP, Nguyen PT, Shoguchi E, Hisata K, Vo TTB, Inoue J, Shinzato C, Le BTN, Nishitsuji K, Kanda M (2018). A draft genome of the striped catfish, *Pangasianodon hypophthalmus*, for comparative analysis of genes relevant to development and a resource for aquaculture improvement. BMC Genomics.

[b29-tlsr-33-2-257] Kumar S, Stecher G, Li M, Knyaz C, Tomura K (2018). MEGA X: Molecular evolutionary genetic analysis across computing platforms. Molecular Biology and Evolution.

[b30-tlsr-33-2-257] Lai PN, Lim LWK, Chung HH (2021). Mutagenesis analysis of ABCB8 gene promoter of *Danio rerio*. Trends in Undergraduate Research.

[b31-tlsr-33-2-257] Lau MML, Lim LWK, Chung HH, Gan HM (2021a). The first transcriptome sequencing and data analysis of the Javan mahseer (*Tor tambra*). Data in Brief.

[b32-tlsr-33-2-257] Lau MML, Lim LWK, Ishak SD, Abol-Munafi A, Chung HH (2021b). A review on the emerging asian aquaculture fish, the Malaysian Mahseer (*Tor tambroides*): Current status and the way forward. Proceedings of the Zoological Society.

[b33-tlsr-33-2-257] Lerebours A, To VV, Bourdineaud JP (2016). *Danio rerio* ABC transporter genes abcb3 and abcb7 play a protecting role against metal contamination. Journal of Applied Toxicology.

[b34-tlsr-33-2-257] Letunic I, Khedkar S, Bork P (2021). SMART: Recent updates, new developments and status in 2020. Nucleic Acids Research.

[b35-tlsr-33-2-257] Lim LWK, Chung HH, Ishak SD, Waiho K (2021a). Zebrafish (*Danio rerio*) ecotoxicological ABCB4, ABCC1 and ABCG2a gene promoters depict spatiotemporal xenobiotic multidrug resistance properties against environmental pollutants. Gene Reports.

[b36-tlsr-33-2-257] Lim LWK, Chung HH, Lau MML, Aziz F, Gan HM (2021b). Improving the phylogenetic resolution of Malaysian and Javan mahseer (*Cyprinidae*), *Tor tambroides* and *Tor tambra*: Whole mitogenomes sequencing, phylogeny and potential mitogenome markers. Gene.

[b37-tlsr-33-2-257] Lim LWK, Tan HY, Aminan AW, Jumaan AQ, Mokta MZ, Tan SY, Balinu CP, Robert AV, Chung HH, Sulaiman B (2018). Phylogenetic and expression of ATP-binding cassette transporter genes in *Rasbora sarawakensis*. Pertanika Journal of Tropical Agricultural Science.

[b38-tlsr-33-2-257] Liu S, Li Q, Liu Z (2013). Genome-wide identification, characterization and phylogenetic analysis of 50 catfish ATP-binding cassette (ABC) transporter genes. PLoS ONE.

[b39-tlsr-33-2-257] Liu X, Li S, Peng W, Feng S, Feng J, Mahboob S, Al-Ghanim K, Xu P (2016). Genome-wide identification, characterization and phylogenetic analysis of ATP-binding cassette (ABC) transporter genes in common carp (*Cyprinus carpio*). PLoS ONE.

[b40-tlsr-33-2-257] Ma X, Shang M, Su B, Wiley A, Bangs M, Alston V, Simora RM, Nguyen MT, Backenstone NJC, Moss AG, Duong TY, Wang X, Durham RA (2021). Comparative transcriptome analysis during the seven developmental stages of channel catfish (*Ictalurus punctatus*) and tra catfish (*Pangasianodon hypophthalmus*) provides novel insights for terrestrial adaptation. Frontiers in Genetics.

[b41-tlsr-33-2-257] Md Yusni NZ, Lim LWK, Chung HH (2020). Mutagenesis analysis of ABCG2 gene promoter of Zebrafish (*Danio rerio*). Trends in Undergraduate Research.

[b42-tlsr-33-2-257] Michalova V, Murray BW, Sultmann H, Klein J (2000). A contig map of the Mhc class I genomic region in the zebrafish reveals ancient synteny. Journal of Immunology.

[b43-tlsr-33-2-257] Mosser J, Douar AM, Sarde CO, Kioschis P, Feil R, Moser H, Poustka AM, Mandel JL, Aubourg P (1993). Putative X-linked adrenoleukodystrophy gene shares unexpected homology with ABC transporters. Nature.

[b44-tlsr-33-2-257] Newman M, Hin N, Pederson S, Lardelli M (2019). Brain transcriptome analysis of a familial Alzheimer’s disease-like mutation in the zebrafish presenilin 1 gene implies effects on energy production. Molecular Brain.

[b45-tlsr-33-2-257] Nguyen TV, Jung H, Nguyen TM, Hurwood D, Mather P (2016). Evalutaion of potential candidate genes involved in salinity tolerance in striped catfish (*Pangasianodon hypophthalmus*) using an RNA-Seq approach. Marine Genomics.

[b46-tlsr-33-2-257] Nongpiur ME, Khor CC, Jia H, Cornes BK, Chen LJ, Qiao C, Nair KS, Cheng C-Y, Xu L, George R (2014). *ABCC5*, a gene that influences the anterior chamber depth, is associated with primary angle closure glaucoma. PLoS Genetics.

[b47-tlsr-33-2-257] Ogasawara F, Kodan A, Ueda K (2020). ABC proteins in evolution. FEBS Letters.

[b48-tlsr-33-2-257] Park HJ, Kim TH, Kim SW, Noh SH, Cho KJ, Choi C, Kwon EY, Choi YJ, Gee HY, Choi JH (2016). Functional characterization of ABCB4 mutations found in progressive familial intrahepatic cholestasis type 3. Scientific Reports.

[b49-tlsr-33-2-257] Pisareva VP, Skabkin MA, Hellen CUT, Pestova TV, Psarev AV (2011). Dissociation by Pelota, Hbs1 and ABCE1 of mammalian vacant 80S ribosomes and stalled elongation complexes. The EMBO Journal.

[b50-tlsr-33-2-257] Popovic M, Zaja R, Loncar J, Smital T (2010). A novel ABC transporter: The first insight into zebrafish (Danio rerio) ABCH1. Marine Environmental Research.

[b51-tlsr-33-2-257] Ren J, Chung-Davidson YW, Yeh CY, Scott C, Brown T, Li W (2015). Genome-wide analysis of the ATP-binding cassette (ABC) transporter gene family in sea lampreys and Japanese lampreys. BMC Genomics.

[b52-tlsr-33-2-257] Stothard P (2000). The sequence manipulation suite: JavaScript programs for analyzing and formatting protein and DNA sequences. Biotechniques.

[b53-tlsr-33-2-257] Sun H, Nathans J (2000). ABCR: Rod photoreceptor-specific ABC transporter responsible for Stargardt disease. Methods in Enzymology.

[b54-tlsr-33-2-257] The UniProt Consortium (2021). UniProt: The universal protein knowledgebase in 2021. Nucleic Acids Research.

[b55-tlsr-33-2-257] Vlaming MLH, Lagas JS, Schinkel AH (2009). Physiological and pharmacological roles of ABCG2 (BCRP): Recent findings in Abcg2 knockout mice. Advanced Drug Delivery Reviews.

[b56-tlsr-33-2-257] Woodward OM, Kottgen A, Kottgen M (2011). ABCG transporters and disease. FEBS Journal.

[b57-tlsr-33-2-257] Yang M, Derbyshire MK, Yamashita RA, Marchler-Bauer A (2020). NCBI’s conserved domain database and tools for protein domain analysis. Current Protocols in Bioinformatics.

[b58-tlsr-33-2-257] Yeaw ZX, Lim LWK, Chung HH (2020). Mutagenesis analysis of ABCB4 gene promoter of *Danio rerio*. Trends in Undergraduate Research.

[b59-tlsr-33-2-257] Yu CS, Chen YC, Lu CH, Hwang JK (2006). Prediction of protein subcellular localization. Proteins: Structure, Function and Bioinformatics.

[b60-tlsr-33-2-257] Zaja R, Popovic M, Lončar T (2016). Functional characterization of rainbow trout (Oncorhynchus mykiss) Abcg2a (Bcrp) transporter. Comparative Biochemistry and Physiology Part C: Toxicology and Pharmacology.

[b61-tlsr-33-2-257] Zhou Y, Ojeda-May P, Pu J (2013). H-loop histidine catalyzes ATP hydrolysis in the *E. coli* ABC-transporter HlyB. Physical Chemistry Chemical Physics.

[b62-tlsr-33-2-257] Zolnerciks JK, Akkaya BG, Snippe M, Chiba P, Seelig A, Linton KJ (2014). The Q loops of the human multidrug resistance transporter ABCB1 are necessary to couple drug binding to the ATP catalytic cycle. FASEB Journal.

[b63-tlsr-33-2-257] Zutz A, Gompf S, Schagger H, Tampe R (2009). Mitochondrial ABC proteins in health and disease. Biochimica et Biophysica Acta (BBA) – Bioenergetics.

