# Supplementary figures and images for: Genome-Wide Identification, Characterisation and Phylogenetic Analysis of 52 Striped Catfish (Pangasianodon hypophthalmus) ATP-Binding Cassette (ABC) Transporter Genes
Source: Trop Life Sci Res. 2022 Jul 15;33(2):257–93. doi: 10.21315/tlsr2022.33.2.12 (PMC9354906; doi:10.21315/tlsr2022.33.2.12)

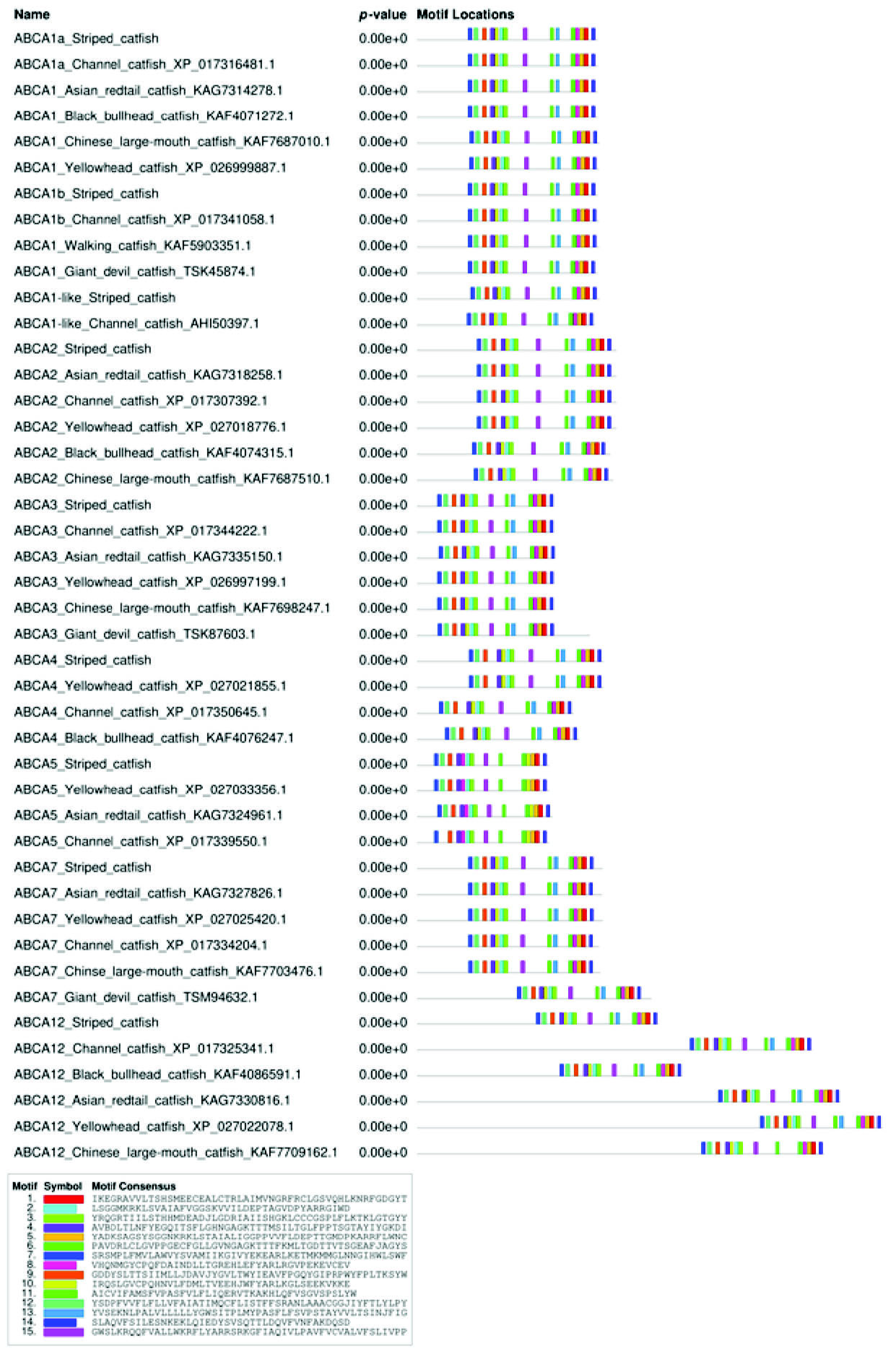

Supplement: Figure S1 — The ABCA subfamily motif analysis. [file TLSR-33-2-257-gS1.tif]

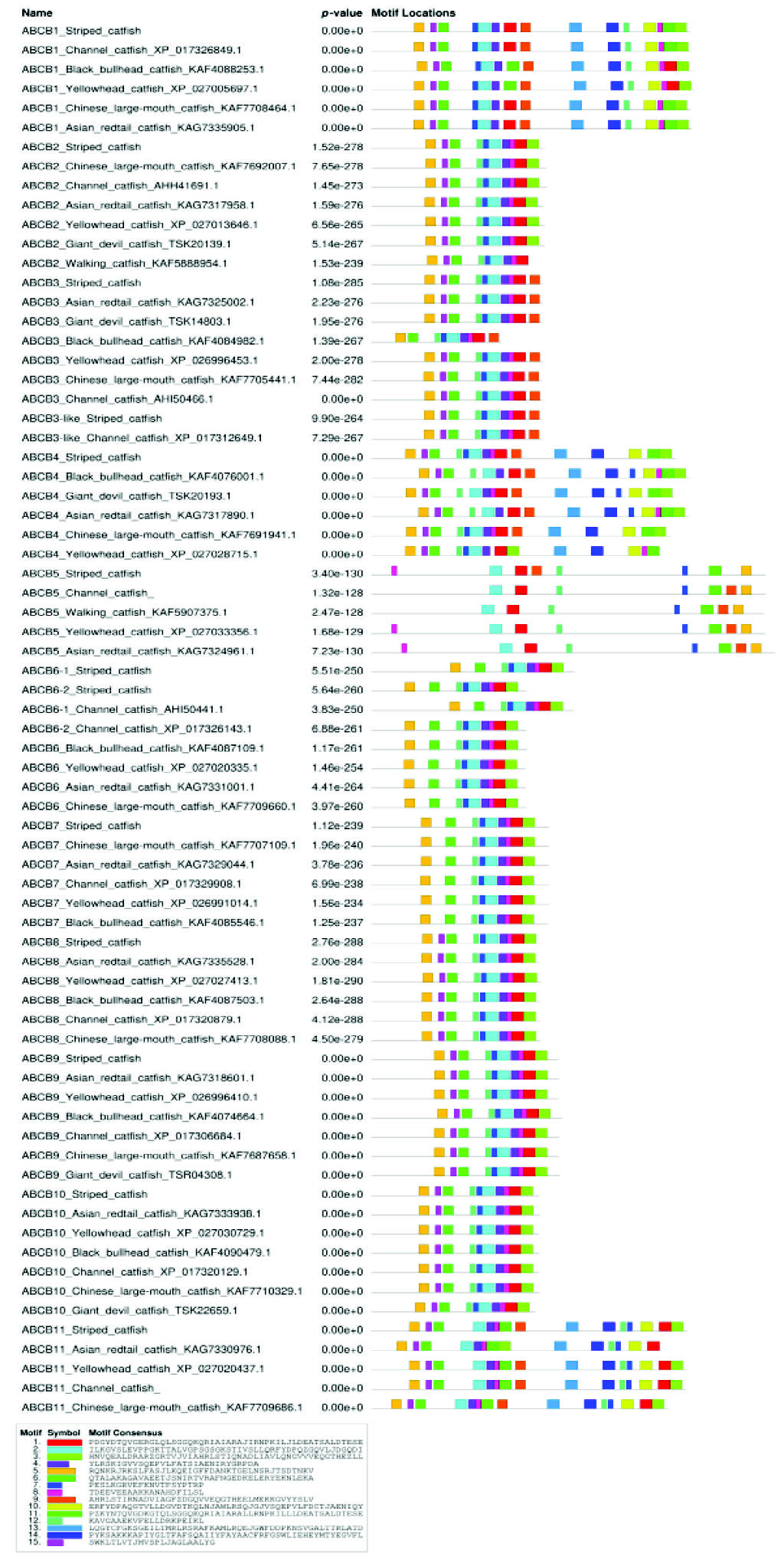

Supplement: Figure S2 — The ABCB subfamily motif analysis. [file TLSR-33-2-257-gS2.tif]

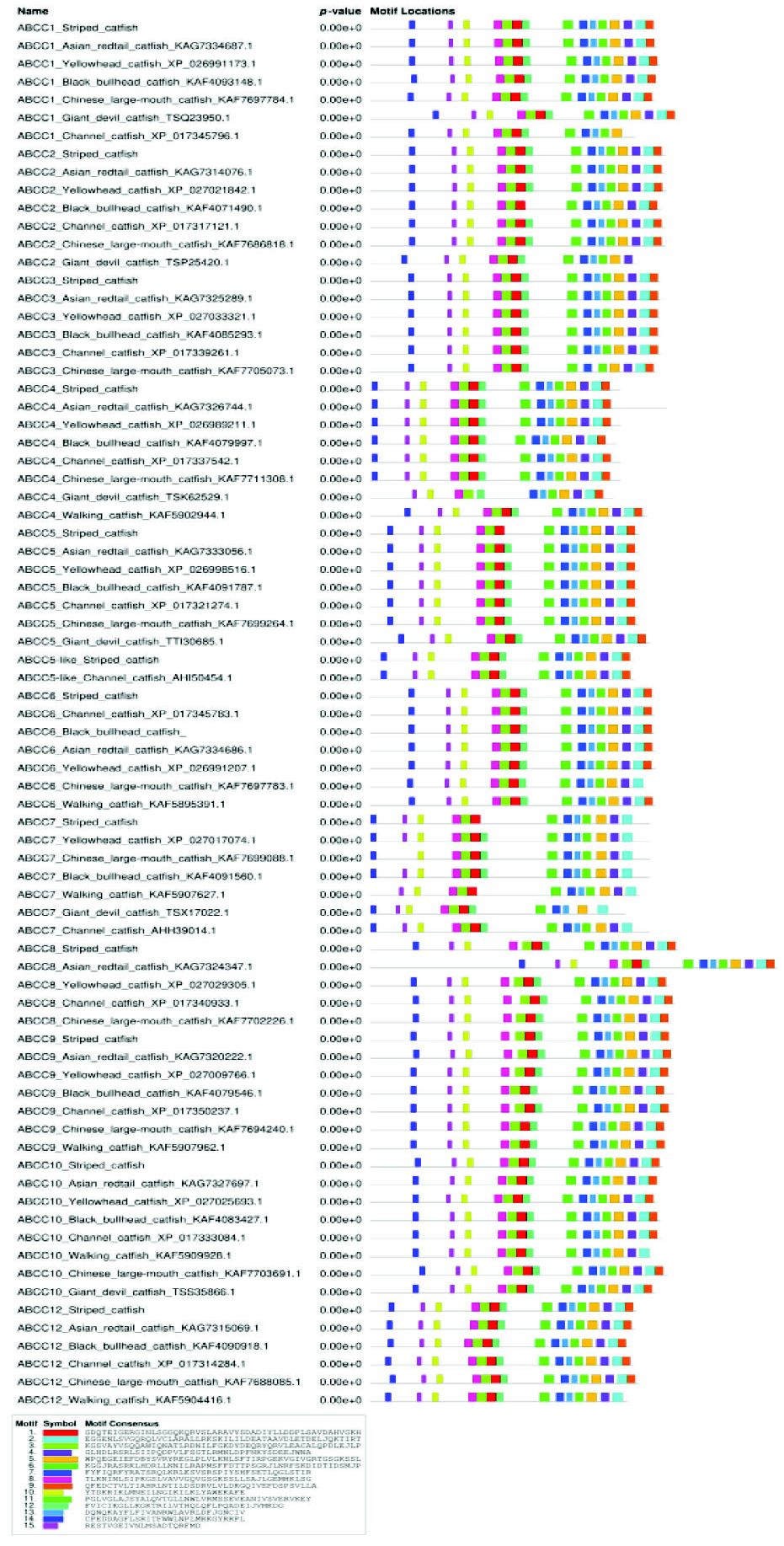

Supplement: Figure S3 — The ABCC subfamily motif analysis. [file TLSR-33-2-257-gS3.tif]

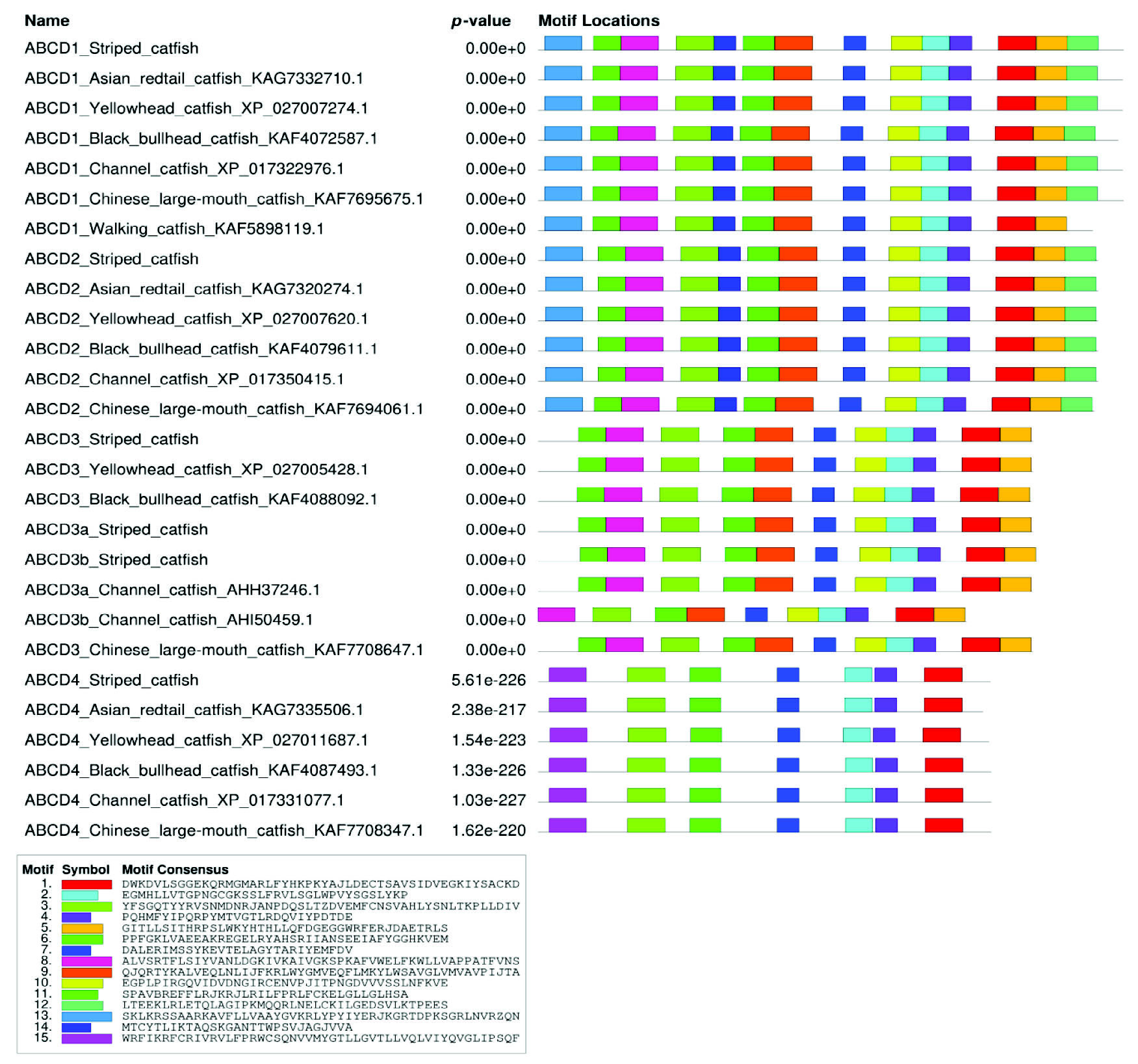

Supplement: Figure S4 — The ABCD subfamily motif analysis. [file TLSR-33-2-257-gS4.tif]

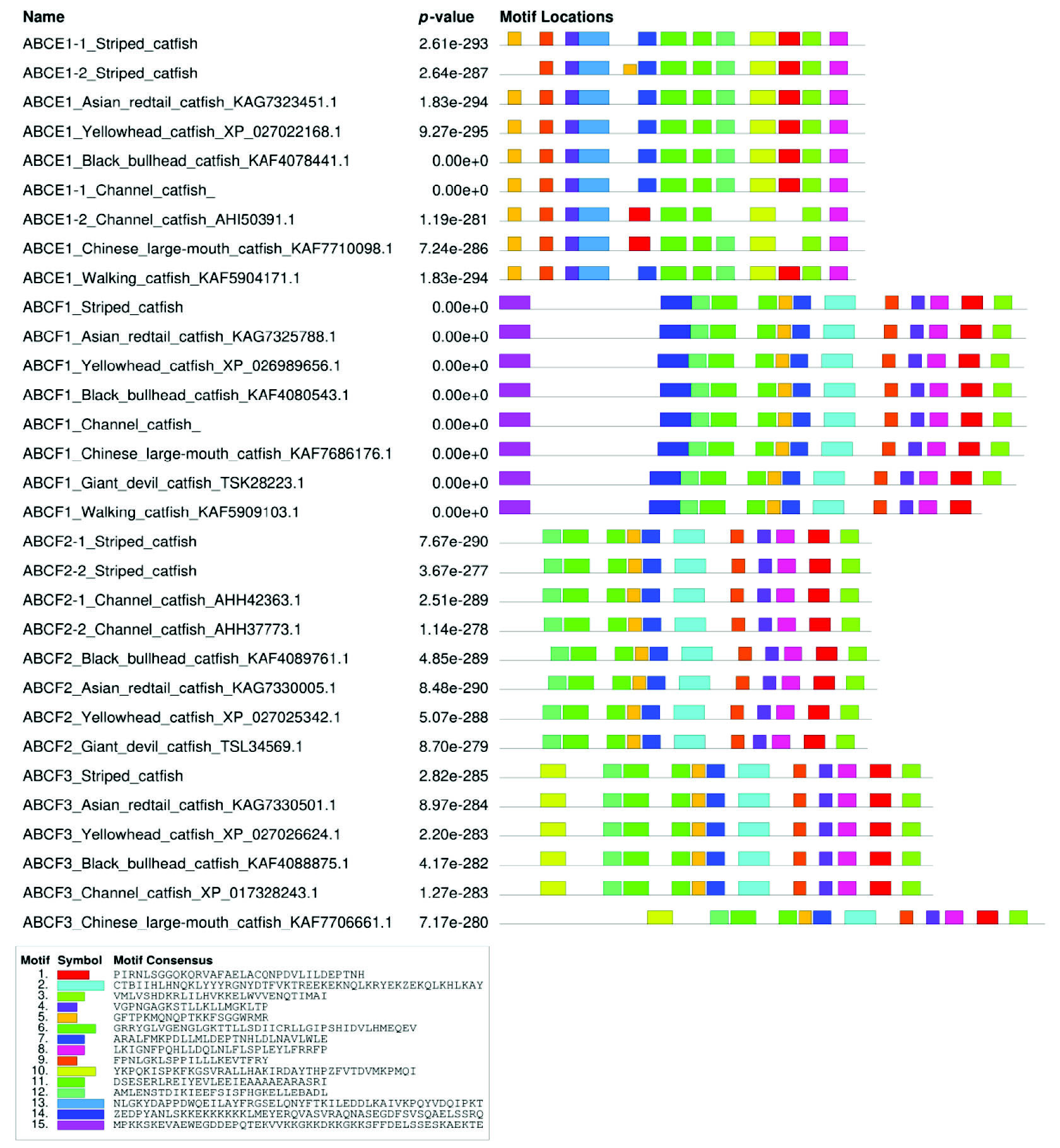

Supplement: Figure S5 — The ABCE-ABCF subfamily motif analysis. [file TLSR-33-2-257-gS5.tif]

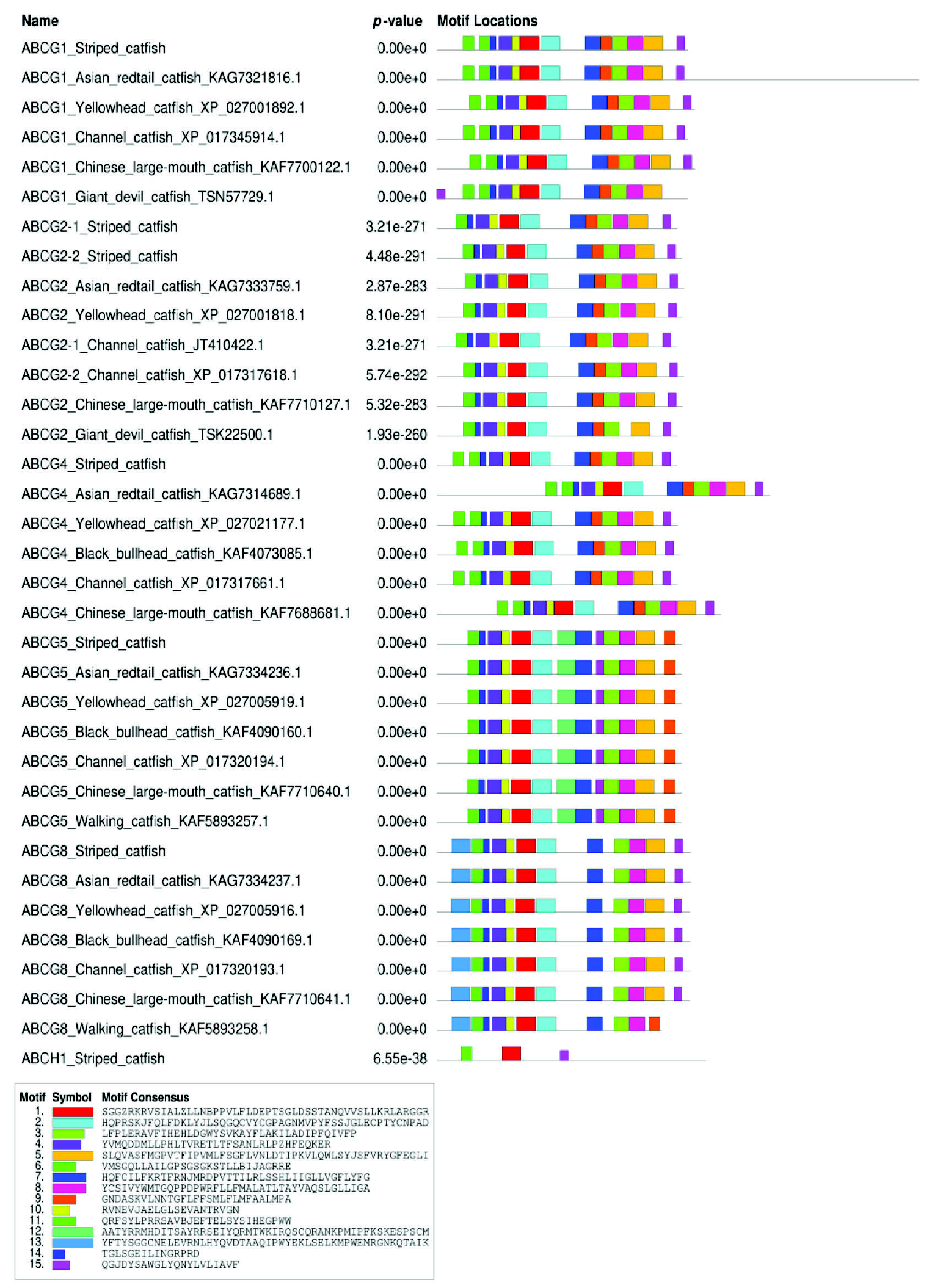

Supplement: Figure S6 — The ABCG-ABCH subfamily motif analysis. [file TLSR-33-2-257-gS6.tif]
